# Antifouling Efficacy on S. epidermidis of Nano-Au Surfaces Functionalized with Polyethylene Glycol (PEG)-Tethered Antimicrobial Peptides

**DOI:** 10.1021/acsabm.5c00253

**Published:** 2025-05-15

**Authors:** Eskil André Karlsen, Mattias Berglin, Adam Hansson, Anders Oskar Lundgren, John S. M. Svendsen

**Affiliations:** † Amicoat AS, Sykehusvegen 23, Tromsø 9019, Norway; ‡ Department of Chemistry, Faculty of Science and Technology, 8016 UiT - The Arctic University of Norway, Tromsø NO-9037, Norway; § RISE Research Institutes of Sweden, Brinellgatan 4, Boras 504 62, Sweden; ∥ Department of Chemistry and Molecular Biology, University of Gothenburg, Gothenburg 40530, Sweden; ⊥ Centre for Antibiotic Resistance Research (CARe), University of Gothenburg, Gothenburg 41346, Sweden

**Keywords:** antimicrobial peptide, antifouling, Certika, ToF-SIMS, contact angle, gold nanoparticle

## Abstract

Cationic antimicrobial peptides (cAMPs) kill bacteria in solution by membrane lysis; however, translating cAMPs into a covalently attached antibacterial coating is challenging since it remains unclear how the specifics of the conjugation impact the antifouling efficacy. Furthermore, studies have typically assessed cAMP coatings with a high and homogeneous surface coverage, although this may be difficult to implement in practice of the materials commonly used in medicine. Herein, we investigate the antifouling efficacy of fractional surface coatings made from poly­(ethylene glycol) (PEG)-tethered cAMPs presented on gold nanoparticles (AuNPs) deposited onto surfaces. For all tested cAMPs, the antifouling efficacy increases exponentially with the 2D surface coverage of the coating. However, although the cAMPs have a similar primary sequence and display similar potency against *Staphylococcus epidermidis* in solution, the cyclic peptide is much more potent after tethering to the AuNPs than the linear counterparts. The attachment of the cyclic cAMPs also led to an unexpected shrinkage of the modified PEG-brush by more than 50%, indicating a restricted mobility of the tethering PEG chains. The shrinkage increased the closeness of the peptide on the AuNP and may thus enable cooperative actions of the grafted cAMPs such as the formation of nanosized peptide clusters that were previously found to enhance cAMP potency in solution. These findings pave the way for antibacterial coatings that cover only a subfraction of a material while remaining active in a clinical setting.

## Graphical abstract

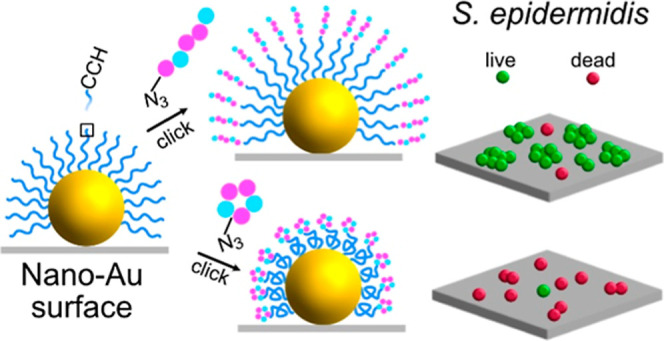

## Introduction

The accelerating global antibiotic resistance crisis poses a serious threat to patient safety.^1^ If this unsettling trend is not mediated urgently, it is estimated that by 2050, up to 10 million humans will ultimately succumb to multidrug-resistant bacterial infections annually.^2^ Infections associated with bacteria and other microorganisms that adhere to and colonize the biomaterial surfaces, a process referred to as biofouling, lead to serious complications in hospitals.^3–5^ Biofouled medical devices are focal points for severe patient infections, thus hurting safety, especially if the bacterium is resistant to conventional antibiotics or if the patient is immunocompromised.^6,7^

One of the pathogens often responsible for infections in healthcare settings is the infamous Gram-positive bacterium Staphylococcus epidermidis,^6,8,9^ a coagulase-negative staphylococcus (CoNS) with high biofilm propensity that belongs to the normal human skin microbiota.^8^ Biofouling by S. epidermidis is associated with infections related to medical devices, a condition that increases treatment cost and can potentially be fatal.^5,6,9,10^ According to a surveillance study, CoNS infections were the culprit of 6.8% of the reported healthcare-associated infections (HAIs), and of these, 47.4% stemmed from S. epidermidis.^11^ Bacterial biofouling of orthopedic implants and catheters by S. epidermidis is a direct cause of HAIs.^11,12^ It is thus imperative to inhibit S. epidermidis from colonizing and proliferating on the surfaces of medical devices. Despite S. epidermidis infections commonly being regarded as treatable, some strains are resistant to various antibiotic groups, including rifampicin.^13^ Rifampicin is commonly used, often in combination with minocycline, as an antimicrobial coating on central-line catheters, whereon its rapid release prevents colonization and subsequent infections.^4,14^ The antifouling efficacy of such coated catheters may be less against resistant strains of S. epidermidis and can even cause rise of new resistance.^15,16^ To use conventional antibiotics as a leaching antifouling ingredient of medical devices should therefore proceed after careful consideration to avoid emergence of antibiotic resistance.

On the other hand, if fouling of medical devices could be reduced by covalently coating the surface with antimicrobial molecules, the resistance selection pressure would greatly be reduced compared to medical devices leaching classical antibiotics. Such a covalent and nonleaching technology would be dependent on active components that can work on the microorganisms without entering the cells. A group of antimicrobial molecules that fulfill such a requirement are the cationic antimicrobial peptides (cAMPs).^17–21^ The main physiochemical characteristic of cAMPs is the amphipathic combination of lipophilic and cationic properties, providing selectivity toward the anionic bacterial cell membranes and a clinically unique mode of action targeting bacterial membranes, and possibly intracellular components, in a manner that make these molecules slower to trigger resistance development.^22,23^ As an example, the polycationic peptide Mel4 has been used as a coating on contact lenses and was clinically evaluated in a phase III trial against corneal infections and inflammations.^24^ Among the properties known to influence the antifouling efficacy of covalently attached cAMPs to surfaces are the intrinsic antimicrobial activity of the peptide, expressed as the minimal inhibitory concentration (MIC), the orientation of the attached cAMP relative to the surface of the coated material, the length and type of the tether used between the surface and the cAMP (allowing room for distance and flexibility), and the surface density of the cAMP.^21,25–28^ However, no comprehensive picture of how these properties are interlinked exists; hence, a good understanding of how to design affective antifouling coatings based on cAMPs is still lacking.

A previous study showed that self-assembled monolayers (SAMs) of cAMPs can greatly reduce the colonization of S. epidermidis on gold (Au) surfaces.^17^ The immobilization of azido-functional peptides in this surface model takes place through a copper­(I)-catalyzed alkyne–azide cycloaddition (CuAAC, colloquially known as a click reaction) to a SAM of alkyne-terminated thiol-PEG molecules on the Au surface, providing a tool to investigate how linker lengths and peptide antimicrobial activity will affect the antifouling efficacy of cAMP coatings. Although the SAM model provides a correlation of the antifouling efficacy of the surface-connected cAMPs in relation to their planktonic antimicrobial activity, the model does not reveal information about other important design elements, such as the influence of the surface coverage of the peptide coating and the peptide density within the coating on the antifouling efficacy. To probe these and similar properties, we propose an enhanced model using gold nanoparticles (AuNPs) attached to a silanized glass substrate as a model (Scheme 1) instead of a homogeneous Au surface.

**Scheme 1:**
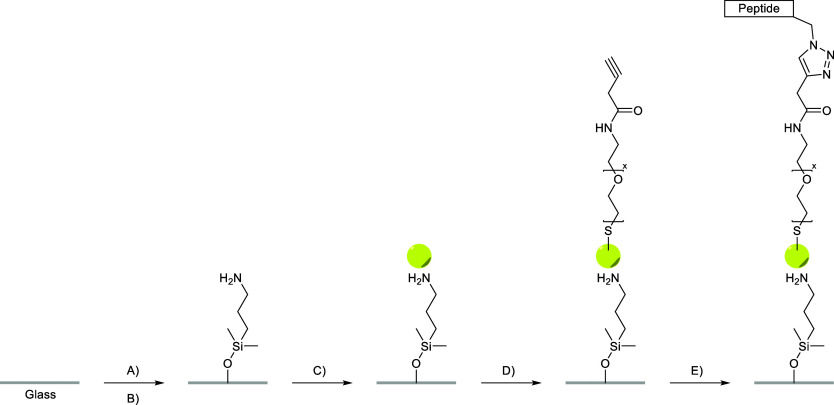
Preparation of the Peptide-Coated Glass Surface Using AuNPs; (A) Glass Slides Were Immersed in 2% Hellmanex III Solution Overnight, Followed by 2M H_2_SO_4_ for 45 min; (B) 3.5% of APDMES for 2 h; (C) X mM Citrate Buffer (*X* = 0.625, 1.25, 10 mM) Supplied with 2.4 nM AuNPs 10 nm indiameter for 60 min; (D) 0.1 mM Alkyne-PEG-Thiol for 2 h; (E) 1:1:1 Ratio of (1) 150 μM Copper­(II) Sulfate Pentahydrate, 750 μM THPTA, and 1 mM Aminoguanidine Hydrochloride, (2) 100 μM Azidopeptide, and (3) 1 mM Ascorbic Acid, for 4 h; Note That the AuNP Is Not Drawn to Scale, Each AuNP Will Bind Hundreds of Alkyne-PEG-Thiols

In this way, the Au surface density is “diluted” relative to the 100% density of the SAM-surfaces. Furthermore, the surfaces are inhomogeneous (“spotted”) in the nanometer realm with the AuNPs presented on a silane-coated surface in contrast to the homogeneous SAM-surface. Since each AuNP has the capacity to bind several peptides, each peptide-functionalized AuNP represents a cluster of peptides. The AuNP model can provide important information about how to best design efficient antibacterial coatings on materials that cannot be completely covered by a monolayer of cAMP.

## Results and Discussion

The surfaces used in this study are covered with AuNPs (10.1 ± 0.7 nm in diameter, Figure S1) at different surface densities. Each AuNP is electrostatically linked to the underlying glass substrate and subsequently functionalized with a heterobifunctional PEG-derived linker moiety. In this model, an α-thio-PEG-ω-alkyne derived from PEG600 (SH-PEG) with a disperse chain length centered on average 13 PEG units is used as the linker moiety. These molecules will bind to the AuNPs with a high grafting density; the footprint of each SH-PEG on the AuNP with 5 nm radius is expected to be below 1 nm.^2,29^ To confirm this, the AuNPs were modified with SH-PEG in solution, and their AuNP radii were measured with dynamic light scattering (DLS) measurements. This showed that modification increased the AuNP radius by 5.5 nm (Figure S8). While the Flory radius of SH-PEG can be estimated to be approximately 1.5 nm, the measured thickness thus shows that the grafted PEG linkers organize as densely packed brushes extending from the AuNP surface.^30^ The alkyne functionality of the linker was used as a surface-attached anchor for a CuAAC (Click) reaction with an azido-functionalized peptide forming an 1,4-disubstituted 1,2,3-triazole linker-cAMP connection moiety.^31^ The PEG-derived linker thus provides a covalent tether between the surface of the AuNP and the peptide, allowing room for limited molecular (lateral) movement, but only from a defined anchoring position.

The cAMPs included in this study are all based on the pentapeptide RBBRF sequence, a classical RW-sequence where the tryptophan (W) residues are replaced with 4,4′-biphenylalanine (B).^32,33^ This substitution enhances the antimicrobial efficacy of the RW-sequence. Peptide **2b** is chosen to investigate the effect of N-terminal tethering to the surface through an additional heterobifunctional PEG (MW 400 Da). Peptide **2c** will probe the effect of conjugation via an additional azidolysine amino acid that furnishes the azide-click functionality positioned at the C-terminus of the peptide, and peptide **2d** is designed to show the effect of cyclizing peptide **2c**, thus reducing the conformational freedom of the peptide (Figure 1).

**Figure 1:**
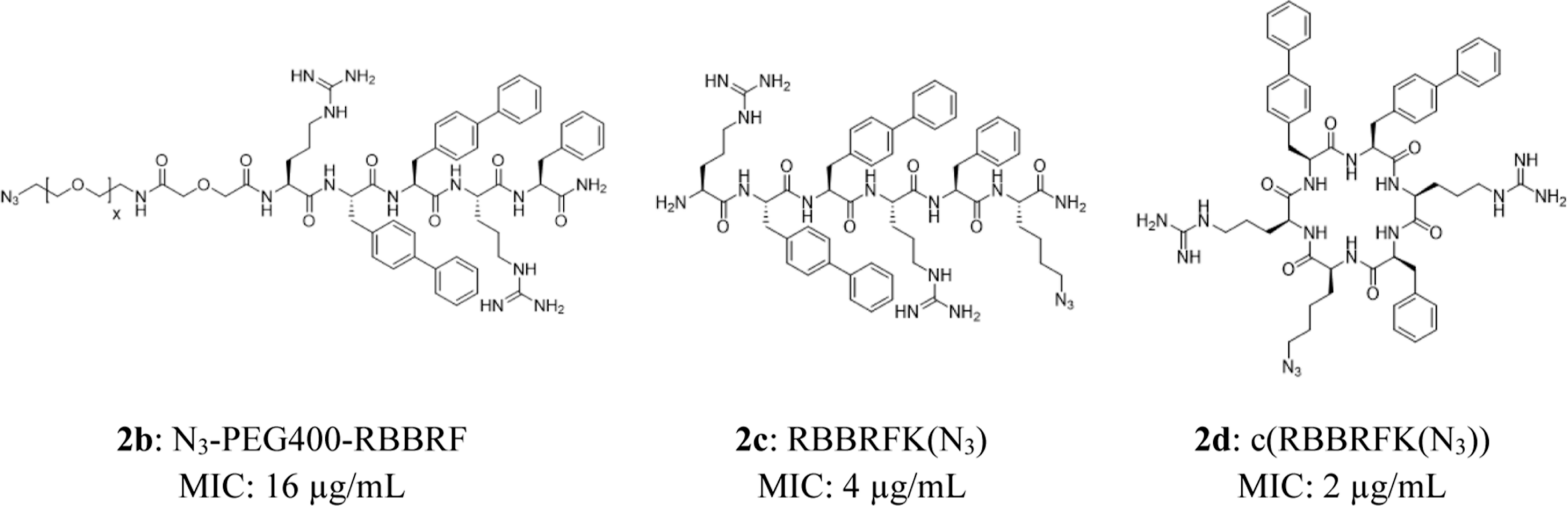
Structure and antibacterial potency of the peptides tested in the study.

All peptides in the library have previously been screened for antibacterial activity against S. epidermidis 1457 in soluble media, as well as the antifouling activity against S. epidermidis RP62A on cAMP-modified SAM-surfaces created on Au-coated SiO_2_ wafers.^17^ While peptides **2c** and **2d** have approximately the same efficacy against planktonic bac (with MIC values of 4 μg/mL and 2 μg/mL, respectively), the PEGylated peptide **2b** is significantly less active (MIC 16 μg/mL). The reduction of efficacy in peptide **2b** is in line with previous observations of the detrimental effect of PEGylation on cAMPs.^34^ When peptide **2d** was connected to a SAM on an Au surface, the antifouling effect was much stronger than for peptide **2b**, which in turn showed a stronger effect than for peptide **2c**.^17^

Compared to the model featuring complete monolayers of cAMPs on SAM-surfaces, the AuNP type of substratum has analytical advantages. The NP-model allows the cAMPs to be presented as a monolayer like on the SAM-surfaces but at a surface coverage depending on the density of AuNPs applied on the substratum, allowing measurements of dose–response curves of surface coating coverage vs antifouling efficacy. Some properties of the surface-bound PEG and cAMPs are also easier to analyze on AuNP-functionalized surfaces than on a planar SAM surface since the effective thickness of a polymer coating on AuNPs in contact with water is reflected by the water contact angle (WCA) of an AuNP-coated surface, in accordance with Cassie’s law.^35^ Furthermore, surfaces displaying only patch-wise (spotty) coverage of antibacterial peptides on an otherwise adhesive and fouling-promoting background is also more interesting from a material application perspective since this type of surface can realistically be applied on “real” materials that otherwise cannot be created with a perfectly homogeneous monolayer coating.

### Preparation and Characterization of AuNP-PEG-Coated Surfaces

The AuNP model surface is created by first silanizing a glass surface with the aminosilane (3-aminopropyl)­dimethylethoxysilane (APDMES). The positively charged amino groups interact readily with the citrate-stabilized negatively charged AuNPs (Figure 2).

**Figure 2:**
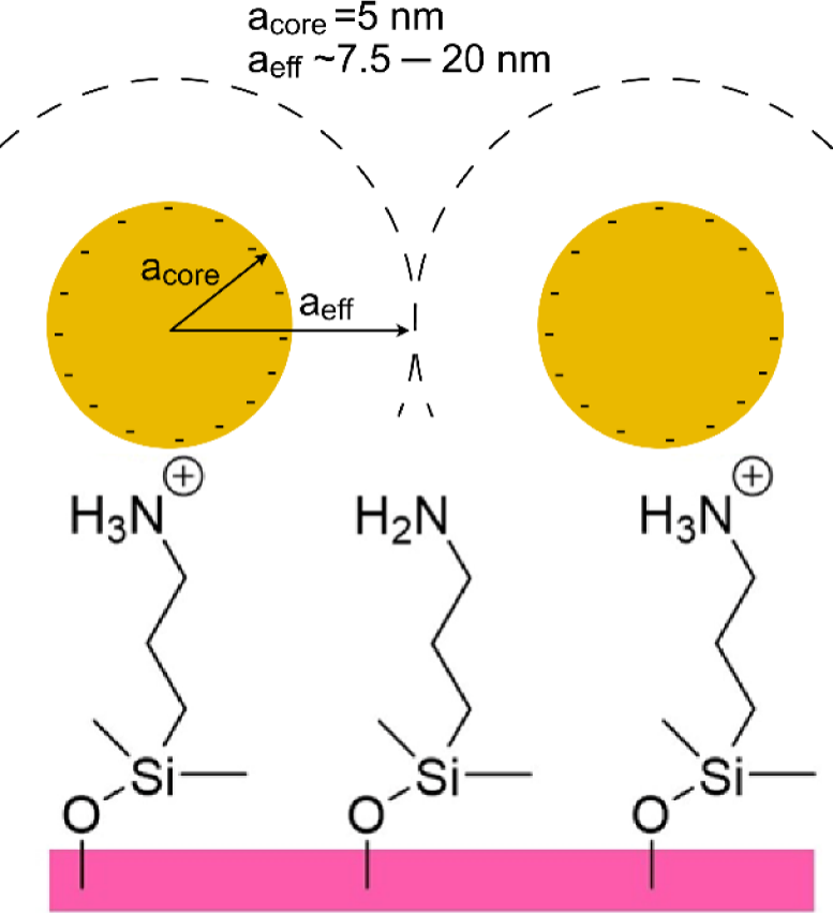
Schematics showing glass slides functionalized with an aminosilanizing agent, (3-aminopropyl)­dimethylethoxysilane, and AuNPs. The AuNPs used in this study have a radius of 5 nm (*a*_core_) and separate on the surface with a typical distance corresponding to the range of their mutual double layer repulsion (*a*_eff_), which decreases with increasing ionic strength of the AuNP solution. Three different buffer conditions were used: 0.625 mM, 1.25 mM, or 10 mM citric buffer at pH = 4.0. Finally, the AuNPs’ surfaces were prepared for the cAMP CuAAC attachment by the binding of the alkyne-PEG-thiol (in the following referred to as SH-PEG).

The connection of AuNPs to the surface is a random sequential adsorption (RSA) process; hence, the projected two-dimensional (2D) AuNP surface coverage at saturation, θ_2D_, would be expected to equal the RSA jamming limit, θ_RSA_ = 54.7%. However, θ_2D_ will usually be lower since the electric double layer repulsion between neighboring AuNPs prevents their close approach during the binding process, and thus, a soft effective AuNP radius, *a*_eff_, can be defined, resulting in a lower effective saturation coverage

1 $$\theta_{2D}=\theta_{RSA}\times {\left(\frac{a_{core}}{a_{eff}}\right)}^{2}$$

The *a*_eff_ can be estimated by calculating the particle–particle interaction potential using the Derjaguin–Landau–Verwey–Overbeek (DLVO) theory.^36^ For AuNPs with a constant surface charge, *a*_eff_ will essentially correspond to the Debye screening length, which gets shorter with increasing ionic strength of the medium. Scanning electron microscopy (SEM) analysis of AuNP surface arrays on conducting materials has shown that for 5 nm radius AuNPs, the minimum effective radius is close to 7.5 nm for a citric buffer concentration of 10 mM. At higher buffer concentrations, the AuNPs quickly aggregate irreversibly.^36^ By decreasing the buffer strength below 10 mM, *a*_eff_ can theoretically become very large; however, in practice, *a*_eff_ will realistically be less than 20 nm since it is difficult to control and maintain low ionic strengths.

Inserting the realistic upper and lower limits of *a*_eff_ into eq 1 shows that for AuNPs with a radius of 5 nm, the θ_2D_ can be varied between 3% and 24% using this method. High surface coverages, approaching the upper limit, have proven particularly easy to reproduce since small variations of the buffer strength around 10 mM have a relatively small impact on the Debye screening length. While analysis of 10 nm AuNPs bound to a nonconducting material like glass by electron microscopy is difficult, the appearance of the AuNP pattern deposited onto semiconducting silicon dioxide can be visualized using SEM. Micrographs of semiconducting silica wafers modified with the same type of silanes as those used for glass followed by 10 nm AuNPs in citric buffer with high, intermediate, and low concentrations are shown in Figures S2–S4. The AuNP-coated glass surfaces to be used in the antifouling efficacy tests were instead thoroughly characterized by time-of-flight secondary ion mass spectrometry (ToF-SIMS) imaging and WCA (ϕ) measurements (Figure 3 and Table 1).

**Figure 3:**
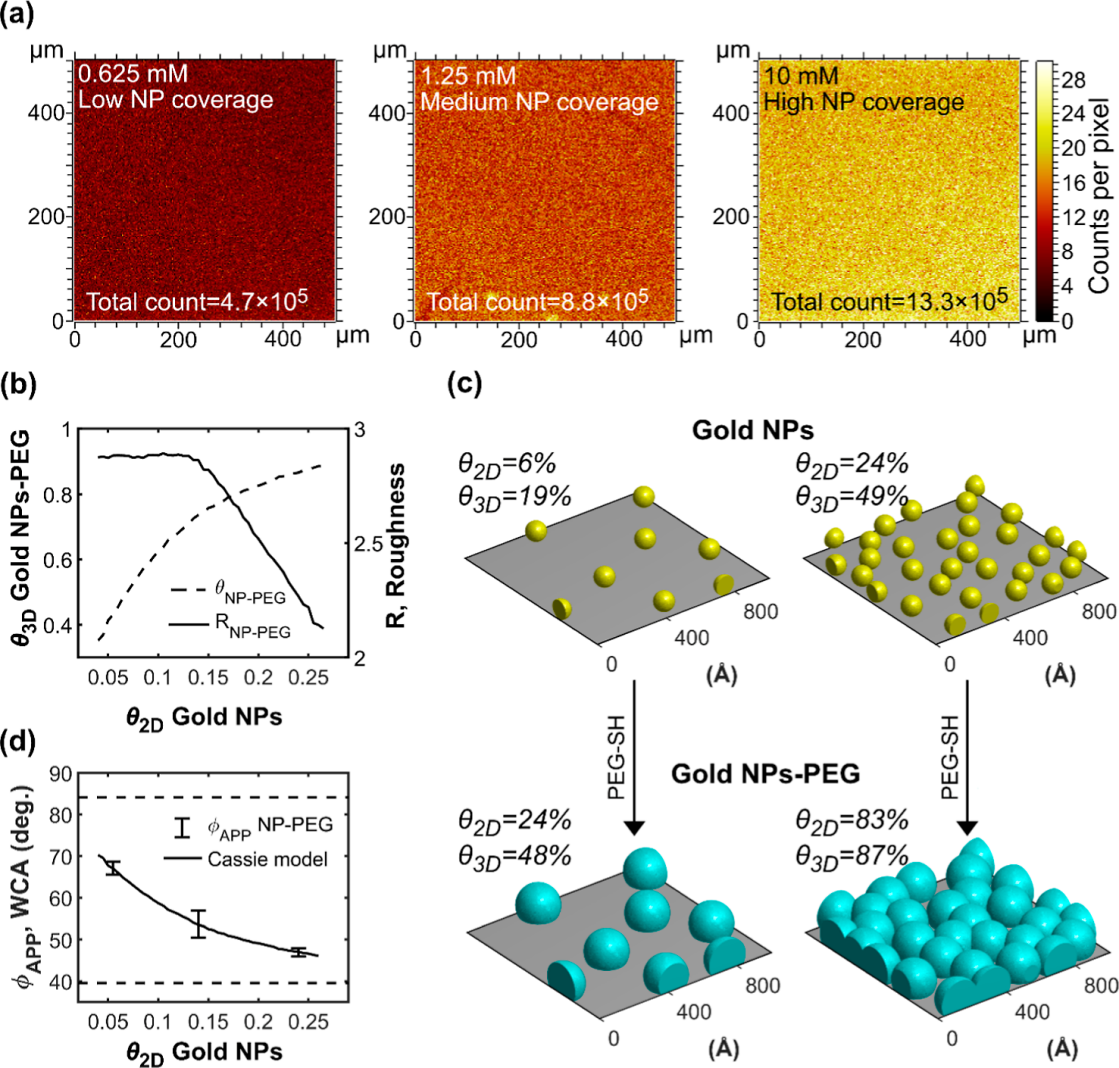
Characterization of AuNP arrays and their modification with SH-PEG. (a) Heat maps showing ToF-SIMS data of AuNPs-functionalized glass surfaces prepared by AuNP binding at 0.625 mM, 1.25 mM, or 10 mM citric acid pH 4. The pixel color represents the summary of ion intensities from Au^–1^ to Au^–10^. (b) Double plot showing how the three-dimensional fractional coverage of PEG-modified AuNPs (θ_3D_, broken line) and the surface roughness (**R**, black line) vary with the two-dimensional projected surface coverage θ_2D_ of AuNPs. The plotted data are the average of ten independent simulations. (c) Snapshots from simulations showing two arrays with randomly distributed AuNPs (θ_2D_ = 6% and θ_2D_ = 24%) before (yellow particles) and after (cyan particles) modification with SH-PEG. (d) The plotted line shows WCAs according to Cassie’s equation for a composite system consisting of PEG-modified AuNPs on a flat silanized surface for different 2D coverages of AuNPs. The broken horizontal lines indicate the contact angles for the extremes of 0% and 100% AuNP-PEG coverage, respectively. The error bars show the SD of the experimental WCAs for PEG-modified AuNP arrays with different coverages, their position on the *x*-axis being the best fit between experimental and calculated data.

**Table 1:** Average (*n* = 4) WCAs ϕ (Standard Deviation within Parentheses) of Different AuNP-PEG-Coated Surfaces Covalently Modified with Antimicrobial Peptides **2b**–**d** ^a^

entry	low coverage (0.625 mM)	medium coverage (1.25 mM)	high coverage (10 mM)
**PEG-2b**	70.2 (1.5)	64.2 (2.6)	52.1 (1.5)
**PEG-2c**	73.3 (1.1)	69.9 (2.3)	57.7 (2.9)
**PEG-2d**	77.0 (3.3)	72.6 (0.9)	60.1 (1.2)
**PEG**	67.1 (1.6)	53.7 (3.2)	47.0 (1.0)

a The control peg is an AuNP-PEG surface treated with peptides without performing the CuAAC reaction and used as a control.

The ToF-SIMS heatmaps shown in Figure 3a confirm the presence of Au on the surfaces and that the amount of Au/area increases with the ionic strength of the citric buffer used when AuNP is dispersed during binding to the surface. The pixel resolution of the ToF-SIMS instrument, which is on the order of 2 μm, does not allow detection of individual AuNPs, but the uniform Au ion intensity when comparing different pixels indicates that the AuNPs cover the surface homogeneously on the micrometer scale. As discussed above, it is reasonable that the total count of Au ions (13.3 × 10^5^) observed for the surface modified in 10 mM citric buffer corresponds to 24% AuNP surface coverage. As the different samples display overall similar surface compositions, we do not foresee any significant matrix effects that could give rise to differences in the ion flux intensities of different chemical functionalities and atoms. Thus, the Au signal is expected to be proportional to the AuNP surface coverage, and the low and medium AuNP coverages would accordingly be approximately 8% and 16%, respectively. WCA measurements were performed too since such experiments were previously shown to successfully estimate the surface density of PEG-modified AuNPs deposited on a hydrophobically modified gold substrates^37^ (Table 1). The WCA is a measure of the wettability of the surface, and the observed angle depends on the intermolecular forces between the molecules exposed on the surface and the water molecules.^38,39^ A large WCA indicates a hydrophobic surface (low surface energy), while a low WCA suggests a hydrophilic surface (high surface energy).^39^ The WCA decreases from ϕ = 67° at low coverage to ϕ = 47° at high coverage; the surfaces modified with AuNP-PEG thus become more hydrophilic with increasing loading. This is expected since the APDMES-modified background surfaces is hydrophobic with a ϕ close to 90° and homogeneous planar Au surfaces modified with SH-PEG representing 100% coverage have a ϕ of 40°.^17,40,41^ Thus, the larger the fraction is of the composite surfaces in contact with water that consist of PEG-coated AuNPs, the closer the ϕ will approach that of the Au surface homogeneously coated with SH-PEG in accordance with Cassie’s law^35^

2 $$\cos\phi_{App}=\theta_{PEG}\cos\phi_{PEG}+\left(1-\theta_{PEG}\right)\cos\phi_{APDMES}$$

where θ_PEG_ is the fractional surface coverage of AuNP-PEG, ϕ_PEG_ is the contact angle of gold completely covered by SH-PEG, and ϕ_APDMES_ is the corresponding contact angle of the background.

Equation 2 can be used to quantitatively assess how the fractional surface coverages of AuNP-PEG (and, further down the line, the AuNP-PEG-cAMP) change by combining the WCA data in Table 1 and the thickness of the SH-PEG coating on the AuNPs’ surfaces. The latter was measured to be 5.5 nm using dynamic light scattering (DLS) of SH-PEG-modified AuNPs in MQ water (Figure S8). Notably, as the AuNP is spheric and large compared to water molecules, the total surface area in contact with water increases for each additional AuNP that is bounded to the surface, and the fractional coverage provided by eq 2 will thus relate to the three-dimensional (3D) surface area of the interface. In the following, θ_3D_ is used to denote the fractional coverage calculated from 3D surface areas, whereas θ_2D_ denotes the projected surface coverage, i.e., how the surface will be experienced by an approaching bacterium. The value of θ_3D_ can be calculated analytically for surfaces sparsely covered by PEG-coated AuNPs; however, for higher coverages, the PEG coating on neighboring AuNPs will overlap and result in more complicated geometries.^37^ The relation between θ_3D_ of AuNP-PEG and θ_2D_ of AuNPs, as well as the surface roughness (*R* = *A*_rea3D_/*A*_rea2D_), for arrays of randomly adsorbed AuNPs was calculated numerically (Figure 3b,c). The θ_3D_ of AuNP-PEG first increases rapidly as a function of the θ_2D_ of the AuNPs, but the increase slows when the surface starts to become crowded with AuNPs. The main reason is clearly understandable from the plot of *R* versus θ_2D_ of AuNPs (Figure 3b, right axis) and from the snapshots from the simulation shown in Figure 3c. If the NPs are isolated on the surface, the additional area (θ_3D_) provided by each AuNP-PEG is close to three times the projected area (note that SH-PEG binding between the AuNP and the surface is limited by the extension of the SH-PEG), but when θ_2D_ > 12%, the additional θ_3D_ area continuously decreases due to the overlap between SH-PEG on different AuNPs. For SH-PEG with higher molecular weight than used here, the overlap between different PEG brushes on surface arrays of AuNPs eventually create an almost flat interface (*R* = 1) that efficiently prevents proteins and cells to bind the surface in-between the deposited AuNPs.^37^ The modeled values of θ_3D_ for AuNP-PEG and the contact angles for flat Au modified with SH-PEG (ϕ_PEG_ = 40°) and glass treated with APDMES (ϕ_APDMES_ = 84°) were inserted into eq 2 to display how ϕ_App_ for the combined system is dependent on θ_2D_ of AuNPs (Figure 3d). A direct comparison between the modeled and experimental WCA data indicates that the lowest surface coverage of AuNPs obtained for 0.625 mM citric buffer is 6% (between 5% and 7%, taking the measured variation in WCA into account) and the medium coverage obtained for 1.25 mM citric buffer is 14% (between 12% and 18%, taking the measured variation in WCA into account). These values are between the coverages predicted by theory (5% and 11%)^36^ and those estimated through ToF-SIMS imaging analysis (8% and 16%). In the following analysis of the antifouling efficacy and antimicrobial peptide binding, these coverage values determined through combination of WCA and the simulated 3D geometries above are used.

### Modification of AuNP-PEG Surfaces with Antimicrobial Peptides

The azido-functional antimicrobial peptides **2b**, **2c**, and **2d** were covalently attached to the terminal alkyne of the SH-PEG on the surface of the AuNPs using the CuAAC reaction (Scheme 1). The formation of covalent bonds between the AuNP-PEG and the antimicrobial peptides was confirmed by specific peptide mass fragments in ToF-SIMS (Figure S5) and localized surface plasmon resonance (LSPR) analysis of peptide binding in the presence or absence of copper ions in the CuAAC reaction. The Tof-SIMS and LSPR data for antimicrobial peptide **2d** are shown in Figure 4, and the corresponding data for the other two peptides are presented in the Supporting Information section (Figure S6).

**Figure 4:**
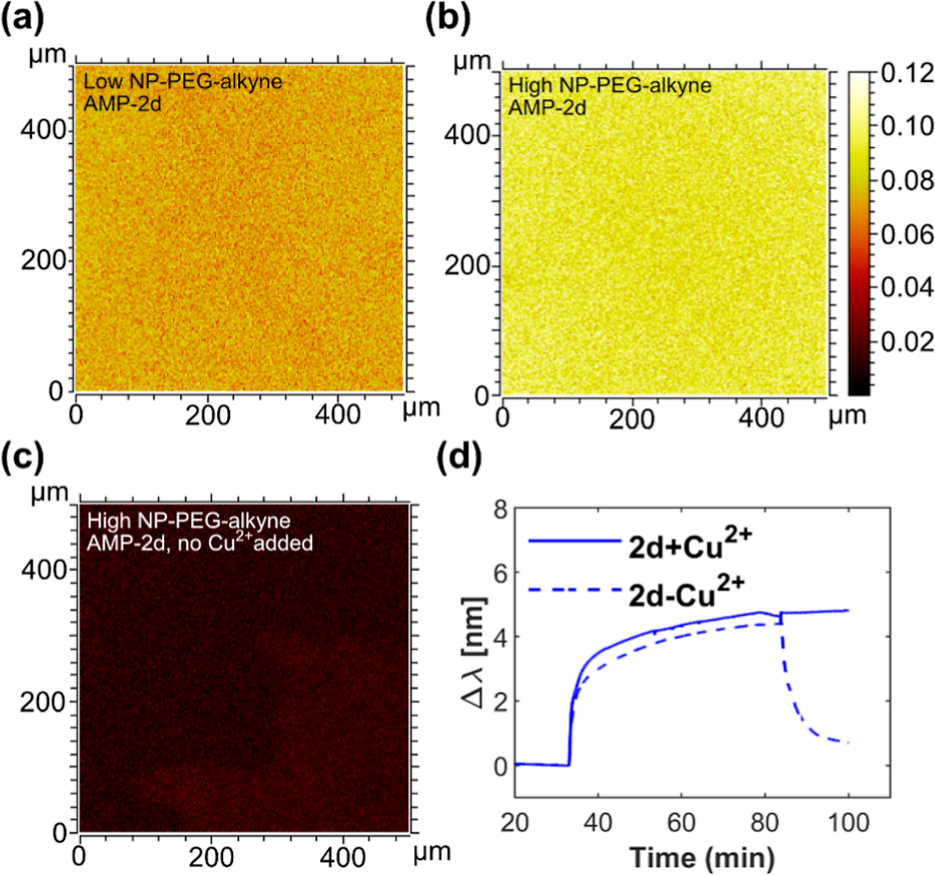
Verification of the covalent binding of antimicrobial peptides to surface-bound AuNP-PEG. The ToF-SIMS heatmaps show the distribution of peptide **2d** bound to glass surfaces coated with (a) low coverage of alkyne-functionalized AuNP-PEG (θ_2D_ = 6%) and (b) high coverage of alkyne-functionalized AuNP-PEG (θ_2D_ = 24%). (c) The ToF-SIMS heatmap shows the result of a control experiment where peptide **2d** was bound to a glass surface with high coverage of alkyne-functionalized AuNP-PEG in the absence of copper ions. The pixel intensities of heatmaps (a–c) represent the summary of ion intensities originating from the cAMPs normalized to the total ion count. (d) The plot shows LSPR binding curves for the injection of peptide **2d** solved in a click reaction solution with or without added copper ions onto Au disks modified with the same alkyne-functional SH-PEG as that used for the modification of the AuNPs.

The ToF-SIMS measurements showed a homogeneous distribution of molecular fragments specific for peptide **2d** on the surfaces modified with high and low coverage of AuNPs (Figure 4a,b). The highest coverage also gave rise to a larger portion of peptide-derived fragments, as expected from peptide-functionalized AuNPs. Similar results were obtained with peptides **2b** and **2c** (Figure S6). Importantly, control surfaces prepared omitting the addition of copper ions in the CuAAC reaction showed very few peptide-specific signals in the ToF-SIMS data (Figure 4c). Similar results were obtained for similar control surfaces made with peptides **2b** and **2c** and control surfaces with low AuNP-PEG coverage (Figure S6). The CuAAC conjugation of the peptides was followed dynamically by LSPR measurements (Figure 4d). The binding curves plotted in Figure 4d show that upon injection over the SH-PEG-modified surface, the peptide accumulates in the proximity of the interface irrespective of the presence or absence of copper ions. However, only in the presence of copper do the peptides remain stably bonded to the surface after rinsing (at approximately 80 min in the plot of Figure 4d). The finding that the CuAAC reaction covalently attaches the peptides to the surface, combined with the efficient wash of noncoupled peptides, means that the antifouling effect of the surfaces can be related to the presence of tethered peptides.

The LSPR binding curves show that the coupling of peptide **2d** to alkyne-functionalized SH-PEG-coated Au surfaces saturates within an hour (Figure 4d). Peptide **2c** coupled even faster, while the peptide **2b** coupled slower, as is expected for conjugation through the azide end group on a PEG400 chain.^42^ The LSPR binding curves for peptides **2b**, **2c**, and **2d** and a thorough discussion about factors underlying different binding kinetics can be found in Figure S7. Peptides **2c** and **2d** had approximately the same LSPR shift at the end of the reaction; however, the shift observed for **2b** was significantly lower. The LSPR wavelength depends on the refractive index in a narrow (a few tens of nanometers) zone right outside of the Au surface, and the sensitivity decreases approximately exponentially with surface separation.^43^ For this multilayer system where peptides were bound on the top of a polymer layer, the interpretation of the shift is ambiguous since the measured shift depends on both the number of bound peptides and the thickness/density of the polymer layer. The footprint of each SH-PEG on the AuNP with 5 nm radius is expected to be about 0.5 nm^2^ in size,^29^ and slightly less than three-quarters of the AuNP’s surface is estimated to be available for SH-PEG binding (Figure 3b). The maximum number of SH-PEGs per AuNP is thus estimated to be 300. Although the current measurements do not reveal the number of peptides is bound at saturation, previous measurements of the WCA for Au-PEG-alkyne SAM-surfaces showed no significant difference between peptide **2b** (ϕ_2**b**_ = 53.5 ± 2.3°), **2c** (ϕ_2**c**_ = 54.9 ± 3.4°), and **2d** (ϕ_2**d**_ = 54.0 ± 2.5°), indicating that the peptide coverage is similar.^17^ The ToF-SIMS data presented in Figure 4a–c (**2d**) and in Figure S6 (**2b** and **2c**) also indicate that peptide coverages on the AuNPs are similar. A possible interpretation of the LSPR data is therefore that the PEG layer between the peptides and the Au surface is thicker for **2b** than for **2c/d**, a reasonable hypothesis given that the structure of **2b** contains an additional PEG400 chain that mediates its binding, extending the total linker length.

To further evaluate the layer thickness issue, the WCAs for the surfaces with different coverages of AuNP-PEG were analyzed in detail after CuAAC peptide connection since, according to Cassie’s law (eq 2) and shown in detail above, the observed contact angle depends on the thickness of the AuNPs coating (Table 1 and Figure 5).

**Figure 5:**
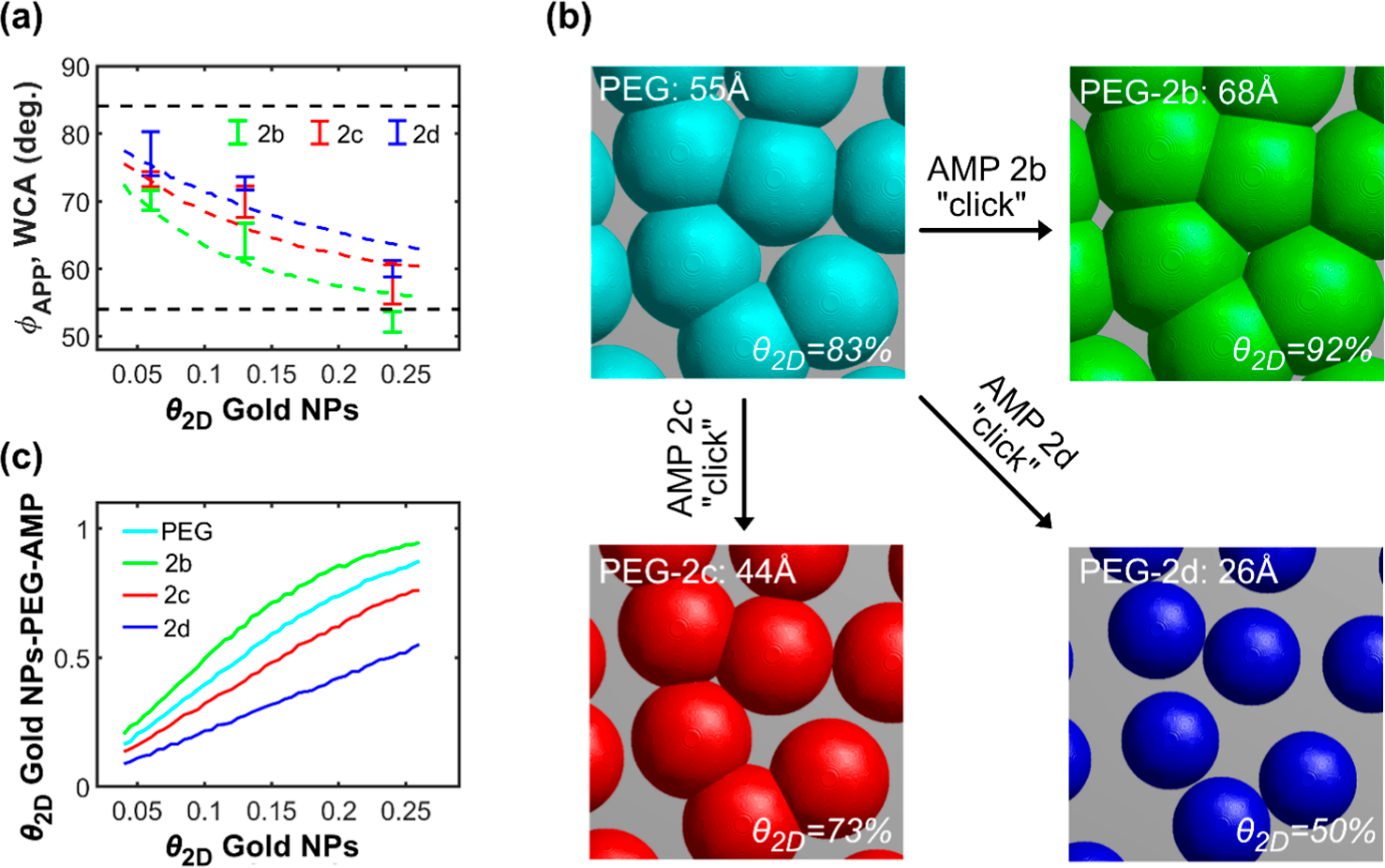
Analysis of WCA data for surfaces with different coverages of AuNP-PEG and different peptide functionalization. (a) The plot shows the experimental WCAs (bars) measured on surfaces modified with different coverages of AuNPs and functionalized with peptides **2b** (green color), **2c** (red color), and **2d** (blue color). The broken colored lines show the best fit of Cassie’s equation (eq 2) to the experimental data corresponding to a coating thickness of 68 Å for peptide **2b**, 44 Å for peptide **2c**, and 26 Å for peptide **2d**. The upper and lower black lines show the WCAs for the limiting situations of a surface without AuNPs and a completely coated Au-PEG-cAMP surface. (b) Snapshot from simulation showing for the same AuNP array with θ_2D_ = 24% the projected 2D surface coverages obtained after SH-PEG binding (cyan) followed by conjugation of peptide **2b** (green), **2c** (red), or **2d** (blue), respectively. (c) The plot shows the predicted projected θ_2D_ of PEG (cyan line) and the projected θ_2D_ of peptides **2b** (green line), **2c** (red line), and **2d** (blue line) as a function of the projected θ_2D_ of AuNPs.

The WCA decreases with increasing AuNP coverage (Table 1 and Figure 5a), as previously observed for the AuNP-PEG SAM-surfaces (cf. Figure 3d). This effect can be interpreted so that for every added AuNP-PEG-peptide, a fraction of the lipophilic background (ϕ_APDMES_ = 84°) is excluded from water contact and replaced by the more hydrophilic PEG-peptide surface of the AuNP (ϕ_cAMP_ = 54°). While the WCAs for Au surfaces completely covered by SAMs of SH-PEG-alkyne and subsequently modified with peptides **2b**, **2c**, or **2d** are the same (within estimated error),^17^ the situation is different for peptide-modified AuNP-PEG where the WCA is higher for **2d** than for **2c**, which again is higher than that for **2b**. The variation of the WCA is in line with the hypothesis discussed above; upon peptide conjugation, the different peptides will give rise to AuNP coatings of different thicknesses, thus excluding varying background surface area per added AuNP. To estimate the thicknesses of the PEG-peptide coatings, Cassie’s law can be used to model the WCA for combinations of θ_2D_ for AuNPs and coating thicknesses as described for the SH-PEG coating (cf. Figure 3). The best fit of the modeled data to experimental WCA is found for a coating thickness of 68 Å for peptide **2b**, 44 Å for peptide **2c**, and 26 Å for peptide **2d**, as shown in Figure 5a,b. In contrast to the fit of WCA to PEG-coated AuNP arrays (cf. Figure 3d), none of the peptides gave rise to an overall perfect fit to the experimental data since for low θ_2D_ of AuNPs, a thinner coating would give a better fit, whereas for high θ_2D_ of AuNPs, a thicker coating would give a better fit. This result shows that the geometrical model that works well for AuNP-SH-PEG might not capture details of the AuNP-PEG-peptide coatings. The AuNP-PEG-peptide coating thickness increases clearly upon binding of **2b** compared to the thickness of the Au-SH-PEG coating alone, while binding of **2c** and, particularly, **2d** causes the AuNP-PEG-peptide coatings to shrink (Figure 5b).

For **2b**, the increased coating thickness is expected as this peptide contains an additional PEG chain of considerable size (400 Da) compared with that of the underlying SH-PEG (600 Da). The decrease in coating thickness observed upon binding of peptides **2c** and **2d** is however surprising. To further investigate this finding, DLS measurements were taken to resolve the effect of peptide binding to the AuNPs modified with SH-PEG-alkyne in solution (Figure S9). These measurements show that while AuNP-PEG remains sterically stabilized in a CuAAC reaction (where the solution have high ionic strength) void of the azidopeptides, the addition of the azidopeptides to the CuAAC reaction immediately destabilizes the particles that start to aggregate. Interestingly, the rate of aggregation follows the sequence observed for the peptide coating thickness, with **2d-**coated AuNPs aggregating twice as fast as AuNPs coated with **2c** and five time faster than AuNPs coated with **2b**. Aggregation due to direct interaction between peptides on different NPs could be excluded since no aggregation was observed upon peptide binding to AuNPs modified with SH-PEG-alkyne of higher molecular weight (3400 Da) extending farther (15 nm) from the AuNP surface (Figure S10). The observation that peptide connection causes the PEG coating to lose its ability to sterically stabilize the AuNPs in solution supports the interpretation of WCA measurements showing that the PEG chains do not extend to the same degree in the presence of the antimicrobial peptides as they do in the presence of pure water. The underlying reason for this phenomenon remains unclear, but among possible explanations are that tethering PEG chains restrict the movement of the peptides, enhancing weak peptide–peptide and/or peptide–PEG interactions within the PEG brush, or that water is prevented from entering the polymer brush, leading to a more closed structure.^44^

Because of the variation in the thickness of the PEG-peptide coatings, the projected 2D coverages of peptides **2b**, **2c**, and **2d** will be very different when applied on an array of AuNPs (Figure 5b). For example, θ_2D,AuNP_ = 24% gives rise to θ_2D,2**b**_ = 92% and θ_2D,2**d**_ = 50% after reacting first with SH-PEG and then with peptides **2b** and **2d**, respectively. This result implies that a bacterial cell that adheres to cAMP-linked AuNP surfaces will experience an almost twice as large contact area with the PEG-**2b** coating than with the PEG-**2d** coating; however, the point density of peptides within the contact zone will be higher for **2d** than for **2b**. Furthermore, as can be seen in the illustration of the modeled data presented in Figure 5b, the coating layer of peptide **2d** never overlaps the AuNP surface layer on neighboring particles, while for peptide **2b**, adjacent layers start to overlap already about 10% AuNP coverage. The projected coverage of **2d** will therefore increase linearly with the AuNP coverage over the full range of possible AuNP coverages, whereas the projected coverage of peptide **2b** is expected to saturate at high AuNP coverages (Figure 5c).

### Antifouling Efficacy of AuNPs Coated with Antimicrobial Peptides

The surfaces coated with AuNPs and the different antibacterial peptides were evaluated for their efficiency in inhibiting the growth of surface-bound S. epidermidis RP62A using the Certika assay originally developed to work for both leaching and nonleaching antibacterial materials (Figure 6).^45^

**Figure 6:**
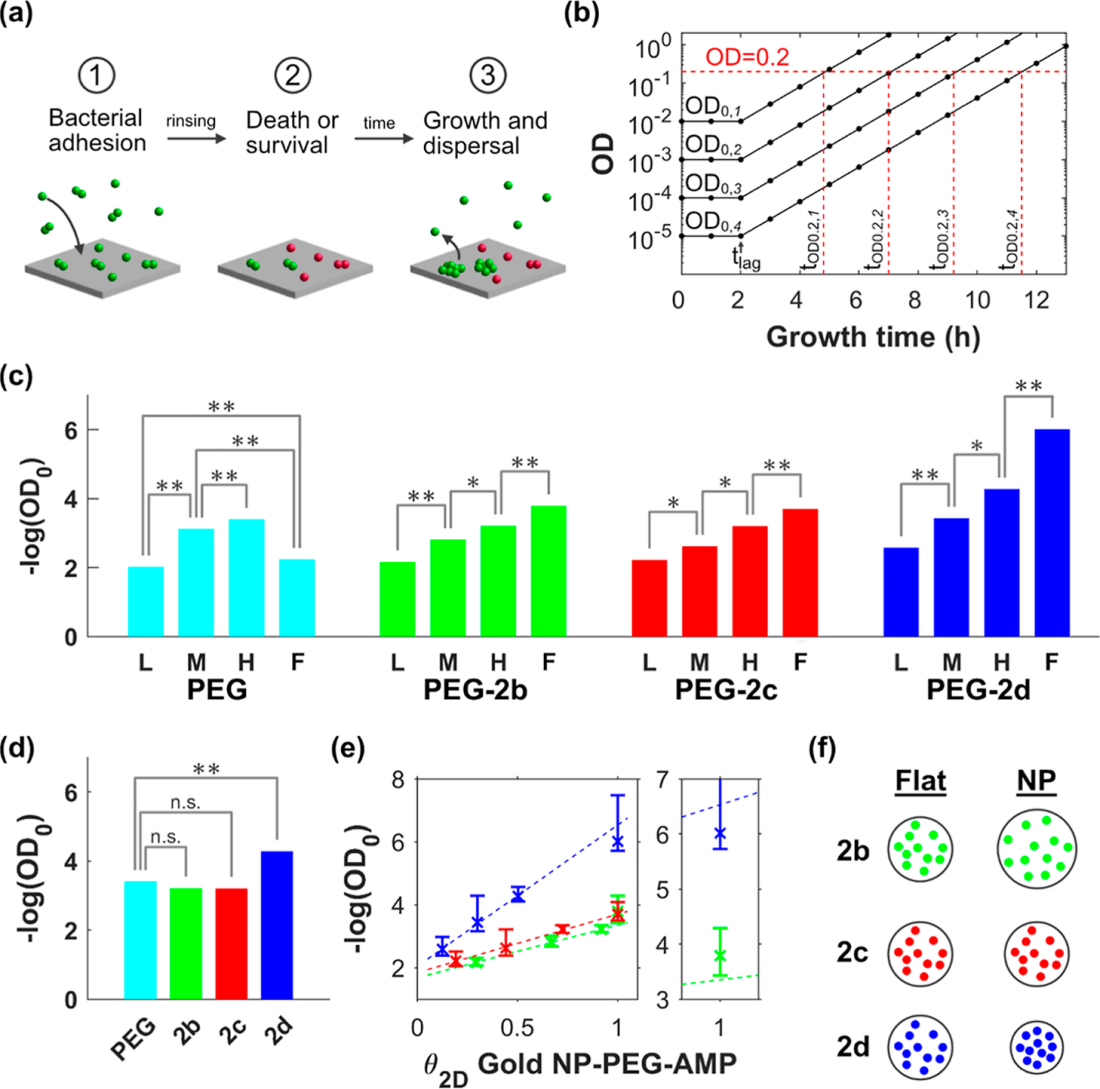
Antifouling efficacy of AuNP surfaces modified with cAMPs. (a) Schematics showing the steps of the Certika assay, green spheres/doublets indicate living bacteria and red spheres/doublets indicate dead bacteria. (b) Theoretical growth curves for samples with OD_0_ between 0.01 and 0.00001 growing with a duplication time (*t*_2_) of 40 min after a lag time (*t*_lag_) of 2 h. OD_0_ is determined for each sample by detecting the time it takes for a sample to grow to an OD value of 0.2 (*t*_OD0.2_) and inserting this value into eq 3 (cf. Experimental Section). (c) The bar plot shows the results of the Certika assay presented as the negative logarithm (log_10_) of OD_0_ for surfaces with different types of coatings (color coded) and projected surface coverage of AuNPs. **L** indicates θ_2D,AuNP_ = 6%, **M** indicates θ_2D,AuNP_ = 14%, **H** indicates θ_2D,AuNP_ = 24%, and **F** indicates values obtained for fully covered SAMs on Au surfaces in a previous study.^17^ Significance was tested using the Student’s *t*-test, * indicates *p* < 0.01, **indicates *p* < 0.001. (d) The bar plot shows a comparison of the results of the Certika assay presented as the negative logarithm (log_10_) of OD_0_ for surfaces with θ_2D,AuNP_ = 24% coated with PEG (cyan), PEG-**2b** (green), PEG-**2c** (red), and PEG-**2d** (blue). Significance was tested and is indicated as in (c). (e) The plot to the left shows the results of the Certika assay presented as the negative logarithm (log_10_) of OD_0_ for surfaces coated with PEG-**2b** (green), PEG-**2c** (red), and PEG-**2d** (blue) as a function of each coating’s projected 2D coverage. The crosses indicate average values and error bars one standard deviation. The broken colored lines indicate the best linear fits to the submonolayer coverages corresponding to different AuNP surfaces. The smaller plot to the right highlights the intersections between the data points for complete monolayers of PEG-**2b** and PEG-**2d** and the linear fits to submonolayer coverages. (f) Schematics describing the relations between exposed contact area and peptide concentration for the different peptide coatings when presented on a flat Au surface and on an AuNP, respectively.

In the Certika assay, bacteria are first incubated with the surface, allowing them to bind to the surface with antibacterial or antifouling properties. Upon binding, a fraction of the bacteria might die. After the first incubation, the surfaces are rinsed, and growth medium is added. The surviving bacterial fraction may however create daughter cells that will be released to the surrounding solution (Figure 6a). By sampling this solution and determining its concentration of living bacteria, we acquired a measure of the ability of the surfaces to inhibit bacterial growth. The bacterial concentration is typically too low to be measured in a classical optical density (OD) measurement so, instead, the samples are put in 96-well plates and allowed to grow until they reach a threshold OD value of 0.2 (Figure 6b). The time required to reach the threshold (*t*_OD0.2_) together with the duplication time (*t*_2_) and the lag time (*t*_lag_) can then be inserted in eq 3 to calculate the initial concentration (OD_0_) for each sample. In Figure 6c, the result of the Certika assay is summarized, presenting the antifouling efficacy of a coating consisting of PEG only and the three peptide coatings PEG-**2b**, PEG-**2c**, and PEG-**2d** on surfaces with low (L), medium (M), and high (H) AuNP coverage, as well as on SAM-surfaces fully (F) covered by the respective peptide coating.^17^ Note that the efficacy is indicated as the negative of the logarithm (log_10_) of OD_0_, and a higher bar thus indicates larger antifouling efficacy. The results show that for all AuNP coatings, including the unmodified PEG, there is a significant efficacy increase in the antifouling effect upon increasing AuNP coverage. However, when comparing the different coatings at the same AuNP surface coverage, only peptide **2d** gives rise to a significantly stronger antifouling efficacy than that of just PEG. This result is highlighted in Figure 6d, presenting the Certika results for the different coatings on surfaces with θ_2D,AuNP_ = 24%. However, when comparing the results for the AuNP coatings with those from our earlier study featuring completely coated Au surfaces, all complete peptide coatings give a higher efficacy than the corresponding highest (H) AuNP-PEG-cAMP coatings, while the Au surfaces coated with just PEG were intermediate between the lowest (L) and medium (M) AuNP-PEG coatings (Figure 6c).

An important difference between using AuNP-PEG arrays or a fully coated Au-PEG SAM-surface to present the antimicrobial peptides is therefore that the AuNP-PEG array seemingly possesses an inherent antifouling capacity that is on par with the efficacy of the peptide coatings **2b** and **2c**. The AuNPs are functionalized (and protected) with the PEG-alkyne linker so a direct antimicrobial effect of the AuNPs themselves is unlikely. It is, however, well-known that surface-grafted PEG can prevent surface binding of proteins and cells, especially if the grafting density is high and the PEG chains are long.^46–48^ The Certika assay cannot discriminate whether the antifouling effect is due to low bacterial adhesion (Figure 6a, step 1), or an antibacterial effect due to contact killing (Figure 6a, step 2). The increased antiadhesive properties of surface-bound AuNP-PEG compared to those of Au-PEG may thus have several causes. Due to the curvature of the AuNPs, the volume segment available for the PEG chains outside the particle’s surface is larger than the corresponding volume next to a flat surface; therefore, the AuNPs can harbor an intrinsic higher grafting density. The overall grafting density may also be higher since the AuNP surfaces are more pristine and defect-free compared to evaporated Au surfaces. Finally, the three-dimensionality of the AuNP-modified surface, where the outermost interface consists of the AuNP-bound PEG brushes, may create a situation where the rather stiff bacterial cells are not able to contact the adhesive lipophilic background in-between the AuNPs. This hypothesis is supported by the observation that already the medium (M) AuNP-PEG coverage provides almost as high antifouling efficacy as the high (H) AuNP-PEG coverage (Figure 6c), although a substantial fraction of the background (50%, cf. Figure 5c) remains unmodified in the (M)-surface.

The magnitude of the antibacterial effect is similar for AuNP surfaces modified with PEG and for AuNP surfaces modified with peptides **2b** and **2c** (Figure 6d). In the latter cases, and for the peptide **2d** coating, this is probably not an antiadhesive effect as it is for PEG, but rather due to contact killing, the binding of the antimicrobial peptides increases the overall lipophilicity and adds positive charge to the surface, both of which promote the adhesion of negatively charged bacteria. Furthermore, the dose–response effect observed for the three peptide coatings is qualitatively similar but qualitatively different from that seen for the PEG coating. The plot in Figure 6e shows how the antifouling efficacy depends on the projected 2D coverage of the peptide retrieved from Figure 5c. This dependence is anticipated as the fraction of the bacteria’s contact area over which the peptides are spread out and presented. For each of the three peptide coatings, the Certika data for the three levels of surface coverage investigated line up perfectly on a straight line in the logarithmic plot, showing that the antibacterial efficacy increases exponentially with the contact area between the bacterium and the coating. The exponent is significantly larger for peptide **2d** than for **2b** and **2c**, for which the exponents are similar, implying that this parameter reflects the difference in intrinsic antimicrobial potency of the surface-conjugated peptides. For the same projected surface coverage (θ_2D_) of peptide-modified AuNPs, the efficacy of the peptide **2b** coating is however about half that of the peptide **2c** coating. It is reasonable to believe that this is because peptide **2b**, when presented on NPs, occupies a larger area than peptide **2c** due to the PEG extension. Each unit area of the peptide **2b** coating will accordingly have a lower peptide density than that of the peptide **2c** coating (Figure 6f).

To fully establish the functional dependence of the antibacterial efficacy on the peptide density, additional experiments featuring surfaces with a fixed coating coverage but varying density of the same peptide are necessary. It is, however, instructive to study the extrapolation of the linear fits to the submonolayer data emanating from the AuNP-modified surfaces to θ_2D,cAMP_ = 1 and compare the intersections with the experimental values for complete monolayer coatings (Figure 6e). While the antibacterial efficacy of the complete monolayer of peptide **2c** can be straightforwardly extrapolated from the submonolayer coverage, the efficacy of the full monolayer coating of **2b** is higher than expected from the extrapolated fit and the efficacy of the full monolayer coating of **2d** is lower than expected from the extrapolated fit. On a fully functionalized Au surface, any change of the PEG tether length upon peptide binding will not influence the density of the peptides; the same number of peptides **2b**, **2c**, and **2d** will on average occupy the same area patch (Figure 6f). We do not know the absolute grafting density of SH-PEG-alkyne on the SAM–Au surfaces or on the AuNPs, but as discussed above, the SH-PEG footprint is likely smaller on the AuNPs for geometrical reasons. The observed differences between the extrapolated and observed activities of the complete monolayer coverages nevertheless indicate that for peptide **2b**, the AuNP coating presents a lower density of peptide to the bacteria than a SAM-Au surface; for peptide **2c**, the density is the same for flat Au and AuNP surfaces; and for peptide **2d**, the peptide density is higher on the periphery of the AuNP coating than on the planar SAM coating. This result indicates that for coatings based on PEG-tethered grafted antimicrobial molecules, especially to NPs and surfaces with high curvature, the length of the PEG tether and any interactions with or within the grafted molecules that impact the ability to extend might have considerable influence on the overall antibacterial efficacy of the surface coating.

In solution, the potencies of linear peptide **2c** and its cyclic version, peptide **2d**, are about the same. However, when surface-tethered via a PEG linker, the potency of **2d** is, however, much higher. The antibacterial effect of tripeptide AMC-109 on S. epidermidis was recently shown to depend on the spontaneous formation of peptide clusters a few nanometers in size.^49^ It can be speculated that both the higher activity of **2d** and its ability to strongly shrink the PEG layer relate to a higher tendency of this peptide to form a similar type of peptide clusters when PEG-tethered, while in solution, the difference between **2d** and **2c** is less pronounced. If such cooperative behavior exists, then there should be a threshold density above which this is possible. The length of the PEG tether should also matter since both very short and very long tethers will lower the chance of peptide–peptide interactions, thus indicating the existence of an optimal intermediate PEG length.

## Conclusions

To improve our understanding about the function of antimicrobial surface coatings based on tethered antimicrobial peptides, model surfaces are needed for which the details about how peptides are presented to the bacteria can be controlled. The reason for this is that most materials are chemically and morphologically too complex to allow an unambiguous molecular-level analysis of the interaction between their surfaces and the bacterial cells. Previous work reported the antifouling efficacy of Au surfaces completely covered with SH-PEG-alkyne onto which different antimicrobial azidopeptides were grafted by means of CuAAC click chemistry. The goal of the present paper is to investigate the efficacy of a similar antibacterial coating that covers only a fraction of a surface since this type of coating could more realistically be applied onto many types of materials without removing or significantly altering other important material parameters.

Model surfaces were prepared by presenting different numbers of nanometer-sized domains of antimicrobial peptides on the top of a lipophilic and positively charged background surface, which in itself can be regarded as promoting fouling. The domains consisted of surface-deposited AuNPs onto which one out of three antimicrobial peptides (**2b**, **2c**, and **2d**) with known antimicrobial activity was grafted via the same SH-PEG-alkyne linker as that previously employed to prepare surfaces with complete monolayer coverages. Interestingly, the binding of the different peptides resulted in different drastic changes of the PEG-tethers; while peptide **2b** itself containing an additional PEG spacer that mediates its binding, as could be expected, gives rise to an overall increase of the PEG layer thickness, both peptide **2c** and especially the most potent peptide **2d** make the PEG layer collapse and shrink. When grafted onto surfaces with the same number of deposited AuNPs, because of the AuNPs’ curvature, peptide **2b** gives rise to coatings with high surface coverage but low density of the presented peptide, while peptide **2d** conversely gives rise to coatings with low overall coverage in the micrometer range but high density of the presented peptide within the nanometer range coated areas. This finding is important since it shows that interactions between a PEG tether and the molecules grafted to it can change the topology of a coating in an unexpected way, influencing its overall activity. Such effects can easily be misinterpreted as due to differences in a peptide’s intrinsic potential.

Taking this effect into account, the antibacterial efficacy of the three different peptide coatings was assessed and analyzed as a function of their surface coverage. This analysis shows that for a constant density of the presented peptide within the coating, the antibacterial efficacy increases exponentially with the fraction of the bacteria being in contact with the coating. The higher potency of the surface-bound peptide **2d** is manifested by a steeper exponential increase than for peptide **2b** and **2c**, which have similar exponents. In contrast to the result obtained for complete monolayer SAM-coatings, the antibacterial efficacy of peptides grafted to AuNPs normalized to the contact area was lower for peptide **2b** than for **2c**, while for peptide **2d**, the area-normalized efficacy of the AuNP coating apparently exceeds that of the flat coating. We suggest that these differences can be explained by the changes in peptide density within the coating that arise as an effect of the PEG extension and collapse, respectively. The current experimental setup, however, does not allow us to establish the functional dependence on how the antibacterial efficacy varies with the peptide density, but this aspect needs to be addressed in further studies. The AuNPs used to create the surface coating in this study are small in comparison to the contact area between the bacteria and the material surface. Thus, future studies will also need to address whether the exponential dependence of the coating’s antibacterial activity on the coverage is similar if the peptides are presented on the surface of larger-sized AuNPs since in the low coverage regime, a single large patch of antimicrobial peptides may be more damaging to the bacteria then several small patches combined.

## Experimental Section

### Synthesis of the Modified PEG-Linker and Azidopeptides

The synthesis of the heterobifunctional azido-PEG-COOH (Mn 400) linker **6b** and the preparation of azidopeptides **2b**, **2c**, and **2d** have been published previously.^17^

### Preparation of AuNP-Peptide Arrays on Glass Surfaces

#### Silanization of Glass Surfaces

The glass slides (18 mm × 18 mm, Zeiss) were washed in Hellmanex III solution and then washed with water and dried under Ar. Sulfuric acid (2M) was added to cover the slides for 45 min on a shaker at room temperature. The slides were then washed thoroughly with water and then with MeOH and finally dried with Ar. Each glass slide was submerged in 1.5 mL of (3-aminoproyl)­dimethylethoxysilane solution (APDMES, 3.5% in MeOH). The submerged slide was put on a shaker and gently stirred for 2 h before washing with MeOH and water and then storing submerged in water in a refrigerator.

#### Assembly of AuNPs and Their Modification with Alkyne-PEG-Thiol

The aminosilanized glass surfaces were added AuNPs using electrostatically limited binding, and subsequently, bound AuNPs were modified with PEG-thiols using the same procedure as that previously used to modify Au surfaces.^37,50^ The AuNPs were prepared according to the method by Slot and Gueze.^51^ The AuNPs were washed twice through centrifugation at 13,000*g*, and pellets were diluted in water to a final concentration of 5 × 10^–8^ M. A solution of 10 mM citrate buffer pH 4 gave concentrations of 1.25 and 0.625 mM. To each container was added 2 mL of citrate buffer (10, 1.25, or 0.625 mM) followed by 100 μL of AuNPs solution (5 × 10^–8^ M). The mixture turned the surfaces pink or light purple, indicating that AuNPs were bound to the surfaces. The mixture was left on the shaker for 1 h before washing thoroughly with water. The surfaces were always kept under the water–air interface and were never allowed to dry. The slides were washed with EtOH before adding 2 mL of 0.1 mM alkyne-PEG-thiol (Mw600, PG2-AKTH-600, Nanocs Inc., NY, USA) ethanolic solution for 2 h. The glass slides were washed repeatedly with EtOH followed by water and stored in a refrigerator immersed in water until use.

#### Covalent Binding of Azidopeptides to AuNP-PEG-Alkyne-Modified Glass Surfaces

The different azidopeptides **2b**, **2c**, and **2d** were bound to the alkynes of the AuNP-PEG-alkyne complexes by means of a CuAAC reaction.^31,52^ Three solutions were made containing (1) 150 μM copper­(II) sulfate pentahydrate, 750 μM tris­(3-hydroxypropyltriazolylmethyl)­amine, and 1 mM aminoguanidine hydrochloride in phosphate-buffered saline (PBS), (2) 100 μM peptide solution in PBS with 1% DMSO, and (3) 1 mM ascorbic acid in PBS. One mL of each solution was added to the container with the slides and was left on the shaker for 4 h. After 4 h, the slides were washed thoroughly with water.

### Surface Characterization Methods

#### WCA Measurements and Data Analysis

The WCA of the glass slides was determined using a Drop Shape Analyzer 100 (Krüss GmBH, Germany). The experiments were carried out under a controlled temperature (22 °C) and at a controlled relative humidity (50%). The syringe filling and positioning of the drop on the surface was performed by the instrument, minimizing manual handling. For the studies, deionized ultrapure water (resistivity >18.2 MΩ× cm) was used as the probe liquid, and images were acquired 10 s after deploying a 5 μL drop on the surface. The contact angle was calculated using the geometrical tangent method assuming an elliptic drop shape.^38^ At least four drops were applied at random locations on each peptide-coated surface or control surface, and the mean values were used for further analysis. The WCA data were evaluated using Cassie’s law for surfaces with heterogeneous chemical composition.^35^ This requires knowledge of the area exposed to water molecules of the coated NPs relative to that of the silane-modified glass background. For high NP coverage, thick coatings lead to complicated 3D geometries since the coating layers of neighboring NPs overlap unpredictably. These areas were estimated numerically using Matlab (MATLAB Version: 24.1.0.2689473 [R2024a] Update 6, The MathWorks Inc., USA) featuring functions of the Image Processing Toolbox. NPs with “hard” core radius (*a*_core_) 50Å and variable effective “soft” diameter (*a*_eff_) that correspond to the extension of different electrostatic repulsions^36^ were positioned in a box with bottom surface dimensions 1500 Å × 1500 Å according to an RSA process until reaching a final coverage of 0.54 of the “soft” spheres. This cutoff was chosen slightly lower than the theoretical RSA limit of 0.547 to speed up the calculations. The surface-bound NPs were then provided with a coating layer extending from the periphery of the NP cores, the thickness (*d*) of which was a selected value between 1 and 100 Å. Note that the NP remains attached to the underlying surface with its core, so that no coating molecules can extend right beneath it at its contact point. Finally, the total area of interface forming between the coated NPs and water, as well as the area of the two-dimensional background silanized glass surface and water, was calculated using the Matlab function “regionprops3”, which estimates the surface area of 3D objects using the Crofton formula and run-length encoding.^53^ The numerical relations between the 3D coverage of coated NPs and the 2D coverage of bound AuNPs (corresponding to the soft sphere diameter) were calculated for a range of coating thicknesses. Each relation was calculated at least 5 times, and the mean values were used as input when applying Cassie’s equation. Snapshots from the simulations were plotted using the Matlab functions “isosurface” and “isocaps”.

#### Verification of AuNPs and Peptide Binding by ToF-SIMS Chemical Imaging

The presence of AuNPs and peptides on the surface after binding was evaluated by measuring the amount of Au atom clusters and peptide-specific molecular fragments released from the surface by the action of a focused ion beam. ToF-SIMS analysis was carried out in a ToF-SIMS IV instrument (IONToF GmbH, Germany), using 25 keV Bi_3_^+^ primary ions at a pulsed current of 0.1 pA (cycle time 150 μs, width 1.2 ns). Each sample was analyzed in the bunched mode at an analysis area of 500 μm × 500 μm with a resolution of 256 px × 256 px. On average, 25 scans were acquired with an acquisition time of 100 s. At least three different positions on the surface were analyzed to detect the eventual inhomogeneities. Reference spectra of pure peptides were measured by placing a drop of peptide dissolved in EtOH at 1% (w/v) concentration on a clean silica wafer. After the evaporation, the peptide was analyzed, and specific mass fragments from each peptide were identified (see spectra in the Supporting Information). The pixel intensities of the ToF-SIMS heatmaps showing cAMP-modified surfaces represent the sum of arginine ions (C_4_H_10_N_3_^+^), 4,4′-biphenylalanine ions (C_13_H_11_^+^), and larger fragments of the whole peptide (e.g., C_57_H_70_N_13_O_6_^+^, C_57_H_70_N_15_O_6_^+^ and C_57_H_69_N_15_O_6_Na^+^). The flux of specific peptide fragments is normalized toward the total ion flux.

#### Time-Resolved Measurements of CuAAC of Peptide Coatings with LSPR Analysis

The peptide coupling to Au surfaces modified with alkyne-PEG-thiol was assessed using an Insplorion S2 instrument (Insplorion AB, Sweden). The sensor chips consisted of embedded Au nanodisk (*r* = ∼60 nm) transducers on a fused silica substrate. The sensor chips were first cleaned by treatment in a UV chamber for 10 min, sonicated for 10 min in 3 mL of EtOH, washed with 3 × 3 mL of water, and dried in a stream of Ar. The sensors were subsequently modified with alkyne-PEG by immersing them in 10 mL of a 1 mM ethanolic solution of HS-PEG-Alkyne (Mw600, PG2-AKTH-600, Nanocs Inc., NY, USA), which was left to incubate overnight at room temperature. Each sensor was thoroughly washed with EtOH (5 × 5 mL) and water (5 × 5 mL) and dried before mounting in a measurement cell. PBS buffer (Gibco, UK) was used as the running buffer, operating at a flow rate of 50 μL/min. After a stable baseline had been reached, 2.5 mL of a freshly mixed CuAAC reaction mixture (cf. CuAAC reaction above) was injected, and the reaction was allowed to proceed for 50 min before switching back to PBS running buffer. The peak of the optical extinction spectrum was determined, and the peak shifts (Δλ) upon physisorption and covalent coupling of the peptide to the alkyne-functionalized AuNPs were recorded with a time resolution of 1 Hz. Data collection and processing were controlled using Insplorer software (ver 1.0, Insplorion AB, Sweden).

#### Measurements of Alkyne-PEG-Thiol Modification and Subsequent Peptide Binding to AuNPs in Solution with DLS Analysis

The AuNPs to be analyzed with DLS were coated with alkyne-PEG-thiols with a molecular weight of 600 or 3400 Da (PG2-AKTH-600 and PG2-AKTH-3400, Nanocs Inc., USA). The SH-PEG was added to AuNPs in aqueous solution to reach a final concentration corresponding to 3000 alkyne-PEG-thiols per AuNP. The AuNP SH-PEG mixture was incubated for 16 h, whereupon nonbound SH-PEG was removed by five sequential filtrations through centrifuge filters (Nanosep Omega 300 kDa, Cytiva, United States of America). The coated AuNPs were gently centrifuged at 4000 rpm for 10 min to pellet larger aggregates formed during the filtration procedure, whereupon the supernatant was used for DLS analysis.

DLS measurements were done using a Zetasizer NanoZS instrument (Malvern Panalytical Ltd., United Kingdom). The sample chamber was set to 25 °C, and the sample was measured consecutively 30 times at a 173° angle; for each data point, data were collected for 1 min. The solvents used were either water for measurements of SH-PEG thickness or a CuAAC solution (see above) for measurements of AuNP-PEG aggregation induced by antimicrobial peptide binding. In the latter measurements, the PEG-coated AuNPs were added first to the click solution contained in a 1 mL cuvette to a final concentration of 3 nM, followed by the addition of copper and finally the different antimicrobial peptides (dissolved in DMSO) to a final concentration of 10 μM corresponding to approximately 10 peptides per available alkyne. A pipet was used to quickly mix the solution, whereupon the cuvette was immediately put into the instrument and the measurements were started.

### Analysis of the Antifouling Efficacy of the AuNP-Peptide Coatings

The antifouling efficacy of different surface subcoverages of AuNP-PEG-peptides assembled onto the silanized glass surfaces was measured with the Certika method^45^ against S. epidermidis RP62A, using the same procedure as that previously used for complete monolayers of the same PEG-peptide coatings.^17^ This method gives a measure of the ability of surface-adhered bacteria to proliferate and release daughter cells into the surrounding bulk liquid. The bacteria dispersed in minimum medium (PBS with 1% tryptic soy broth [TSB]) at a concentration of 1 × 10^6^ colony-forming units per mL were added to wells containing sample surfaces, whereupon these were incubated at 37 °C for 1 h to allow bacteria to adhere. Loosely bound bacteria were then removed by washing the surfaces four times with PBS, whereupon the samples were incubated in minimum medium at 37 °C for 18 h. After the removal of the test samples, each well was supplemented with TSB complete medium. The bacterial growth of the released daughter cells at 37 °C was recorded for a period of 48 h by OD measurements in a microtiter plate reader (BioTek Instruments, Software KC4 ver. 3.4) at a wavelength of 578 nm. The time, *t*_0.2_, needed for bacteria in each well to multiply to reach OD = 0.2 was measured. Assuming that bacteria grew exponentially with doubling time *t*_2_ starting at the end of the lag time, *t*_lag_, to this concentration (OD = 0.2), the concentration of released bacteria, OD_0_, was calculated through

3 $$\log OD_{0}=\log0.2-\log2\times \frac{t_{0.2}-t_{lag}}{t_{2}}$$

In each experiment, *t*_lag_ and *t*_2_ were determined by analyzing the growth curves corresponding to wells containing control samples with known concentrations of bacteria within the interval of OD_0_ 10^–5^ to 10^–2^. The surfaces used in this study had 3.2 times larger area than the Au surfaces used in our previous study^17^ and thus harbored proportionally more bacteria. Therefore, when the outcomes of the Certika experiments were compared, *t*_0.2_ values obtained in the previous study were adjusted by subtraction of the growth time corresponding to a multiplication factor of 3.2 with the given growth rate.

## Supplementary Material



## Notes

TEM micrograph showing the size of the AuNPs used in the study; SEM micrograph showing AuNPs bound to silicon dioxide under high ionic strength conditions; SEM micrograph showing AuNPs bound to silicon dioxide under medium ionic strength conditions; SEM micrograph showing AuNPs bound to silicon dioxide under low ionic strength conditions; (PDF)
