# Supplementary material for: Antifouling Efficacy on S. epidermidis of Nano-Au Surfaces Functionalized with Polyethylene Glycol (PEG)-Tethered Antimicrobial Peptides
Source: ACS Appl Bio Mater. 2025 May 15;8(6):4870–83. doi: 10.1021/acsabm.5c00253 (PMC12175129; doi:10.1021/acsabm.5c00253)
Supplement: Supplementary file 1 [file mt5c00253_si_001.pdf]

Supporting Information

Antifouling Efficacy on *S. epidermidis* of Nano-Au Surfaces Functionalized with Polyethylene Glycol (PEG)-Tethered Antimicrobial Peptides

Eskil André Karlsen [1,2], Mattias Berglin [3], Adam Hansson [3,4,5], Anders Oskar Lundgren [4,5], and John S. M. Svendsen [1,2,*]

[1] Amicoat AS, Sykehusvegen 23, 9019 Tromsø, Norway; eskil.a.karlsen@uit.no

[2] Department of Chemistry, Faculty of Science and Technology, UiT - The Arctic University of Norway, NO-9037 Tromsø, Norway

[3] RISE Research Institutes of Sweden, Brinellgatan 4, 504 62 Borås, Sweden; mattias.berglin@ri.se

[4] Department of Chemistry and Molecular Biology, University of Gothenburg, Gothenburg 40530, Sweden; anders.lundgren@gu.se, adam.hansson@gu.se

[5] Centre for Antibiotic Resistance Research (CARe), University of Gothenburg, Gothenburg 41346, Sweden

* Correspondence: john-sigurd.svendsen@uit.no

Contents

Electron micrographs of AuNPs and AuNP patterns..**p.S2**

Time-of-Flight Secondary Ion Mass Spectroscopy (ToF-SIMS) chemical imaging for the detection of peptide fragments on glass surfaces...**p.S5**

Localized Surface Plasmon Resonance (LSPR) for dynamic analysis of peptide attachment to Au-nanodisc functionalized with PEG-alkyne...**p.S7**

Dynamic light scattering (DLS) analysis of AuNP modifications with SH-PEG-alkyne and binding of cAMPs... .**p.S9**

Electron micrographs of AuNPs and AuNP patterns

Transmission Electron Microscopy (TEM) was used to determine the size of synthesized AuNPs. AuNPs were synthesized according to the protocol detailed in the method section of the main article text, washed once with MQ water and then dispersed in a citric buffer (1.25 mM, pH4.0). A silicon dioxide coated TEM grid (SF300-CU, Electron Microscopy Sciences) was first treated in a UV/ozone chamber for 15 minutes and then modified with APDMES from gaseous phase. This was done by placing the grids in a closed container together with a small amount of APDMES solution (50% in Methanol) for 30 minutes whereupon the grids were washed first with methanol, then with water, and finally dried under N_2. AuNPs were allowed to bind to the modified grid by placing it up-side-down on a droplet of AuNP solution for 30 minutes whereupon it was moved to a droplet of MQ water to remove non-bound AuNPs. Electron micrographs were captured using a FEI Tecnai G2 microscope operated at 160 kV acceleration voltage. AuNP diameters were estimated by manual measurements using the freeware ImageJ. These measurements (n=62) showed that the median and mean diameters of the AuNPs were 10.1 nm (Figure S1), and the size distribution measured as the standard deviation of the AuNPs' diameters was 0.7 nm.

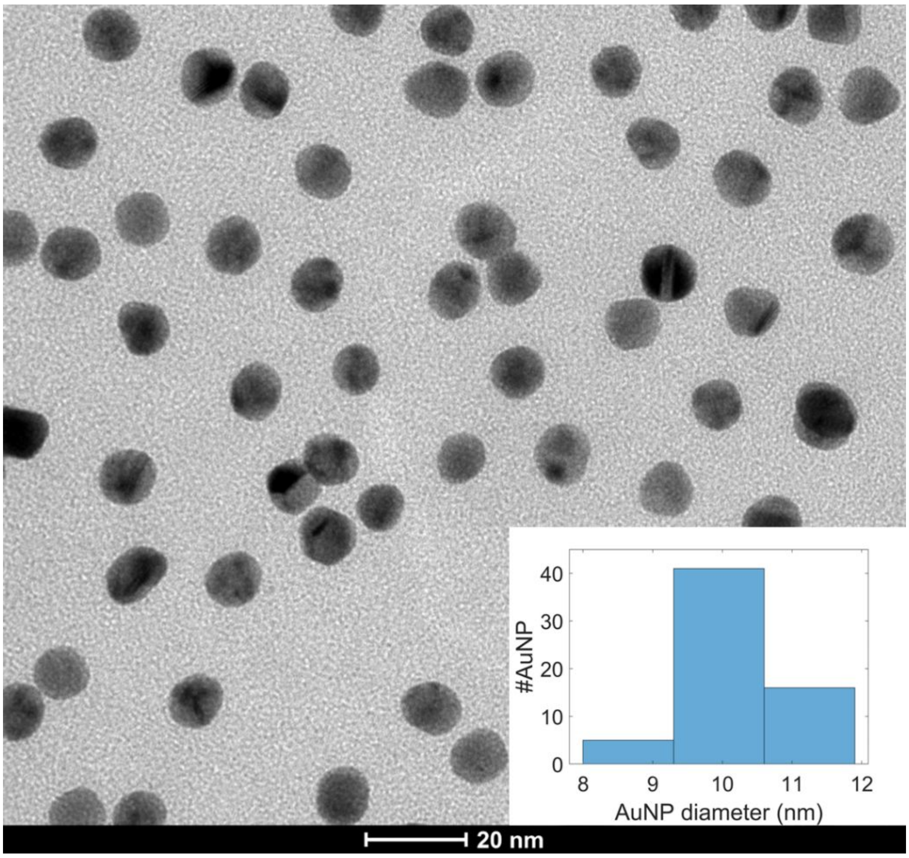

Figure S1: The TEM micrograph display AuNPs synthesized according to the protocol detailed in the method section of the main article text. The inset histogram shows the size distribution (AuNP diameters) of the particles visible in the micrograph.

Scanning Electron Microscopy (SEM) was used to image patterns of 10 nm AuNPs formed by electrostatically limited adsorption to semi-conducting silicon dioxide surfaces modified with

positively charged amino silanes. Gold nanoparticles were synthesized and treated as detailed in the method section of the main article text. P-doped silicon dioxide wafers were cut into pieces of 10x10 mm. These surfaces were then washed and modified first with APDMES and then with AuNPs following the same protocol as used for the glass substrates described in the main article text. Substrates were imaged using a Zeiss Ultra 55 FEG (Figure S2) or a Zeiss 982 Gemini (Figure S3 and Figure S4) scanning electron microscope operating at 5kV in secondary electron mode using an in-lense detector.

The 2D surface coverages of AuNPs were estimated by manually counting the number of particles present within a certain surface area. The areas covered by AuNPs were obtained by multiplying their number with the projected area of a single AuNP with radius 5 nm. This way the highest surface coverage was estimated to 23-24% (Figure S2), the intermediate surface coverage to 16% (Figure S3) and the lowest surface coverage to 7-8% (Figure S4).

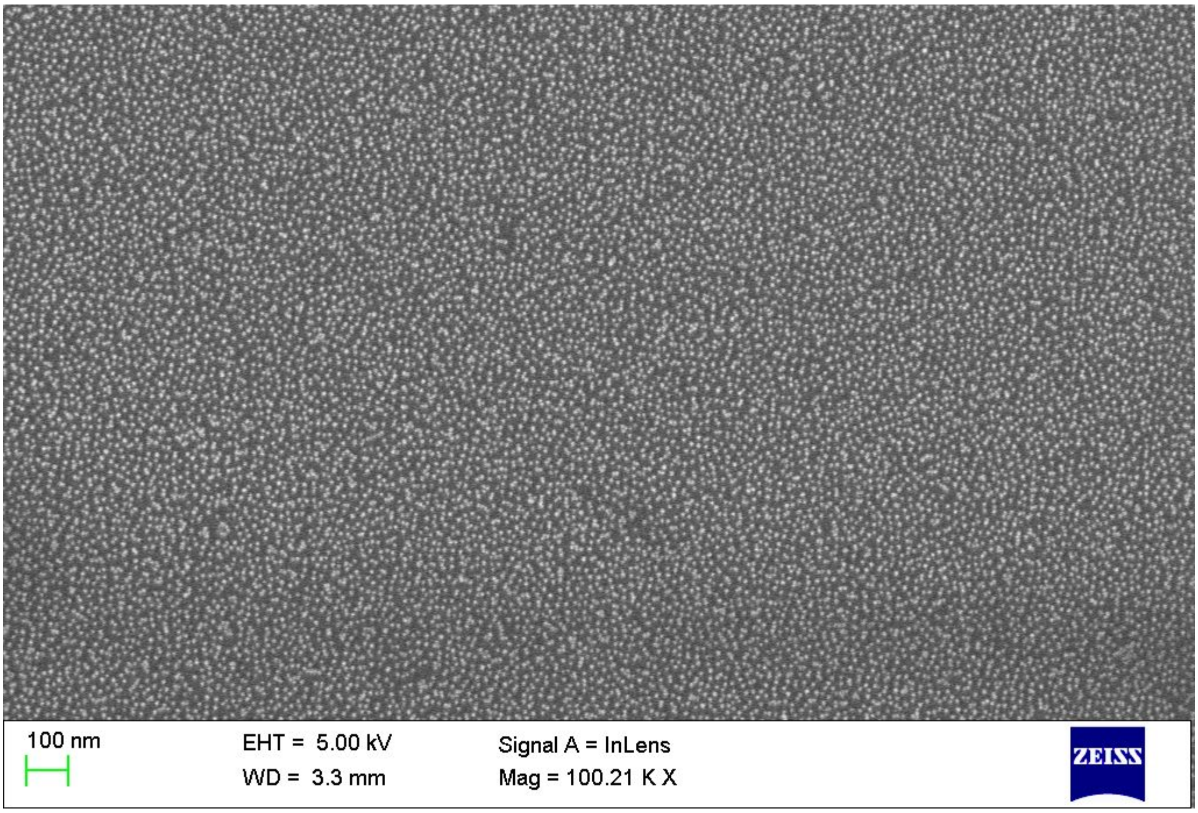

Figure S2: The SEM micrograph displays 10 nm AuNPs bound to a semi-conducting silicon dioxide surface under high ionic strength condition as detailed in the main article text. The AuNP 2D surface coverage was estimated to 23-24%.

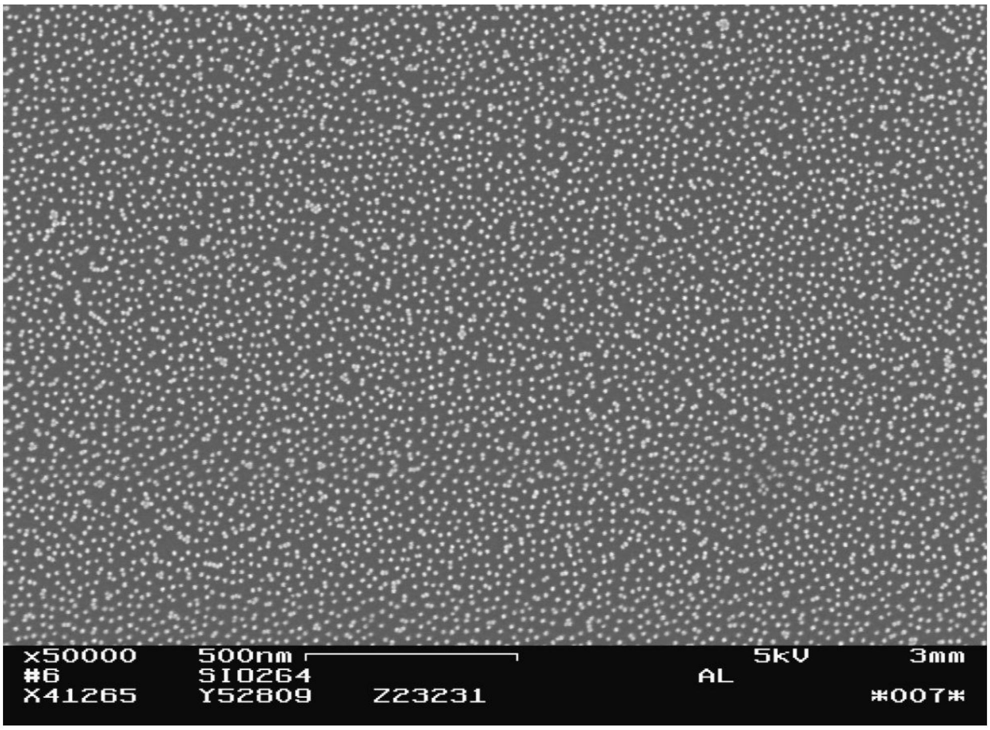

Figure S3: The SEM micrograph displays 10 nm AuNPs bound to a semi-conducting silicon dioxide surface under intermediate ionic strength condition as detailed in the main article text. The AuNP 2D surface coverage was estimated to 16%.

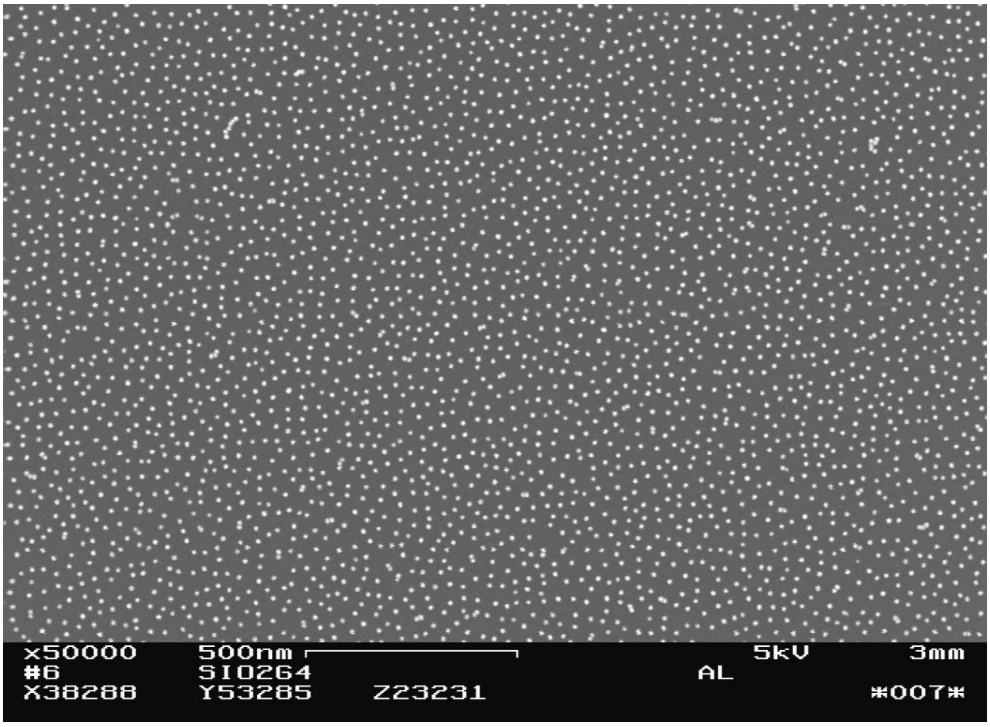

Figure S4: The SEM micrograph displays 10 nm AuNPs bound to a semi-conducting silicon dioxide surface under low ionic strength condition as detailed in the main article text. The AuNP 2D surface coverage was estimated to 7-8%.

Time-of-Flight Secondary Ion Mass Spectroscopy (ToF-SIMS) chemical imaging for the detection of peptide fragments on glass surfaces.

The presence of peptides **2b**, **2c** and **2d** after coupling to NP-PEG-alkyne functionalized glass was investigated by detection of peptide-specific mass fragments. These fragments were identified through measurements of reference samples obtained via deposition of non-conjugated cAMPS on silica via evaporation of an ethanol solution. Examples of mass fragments from respectively peptide are shown in Figure S5.

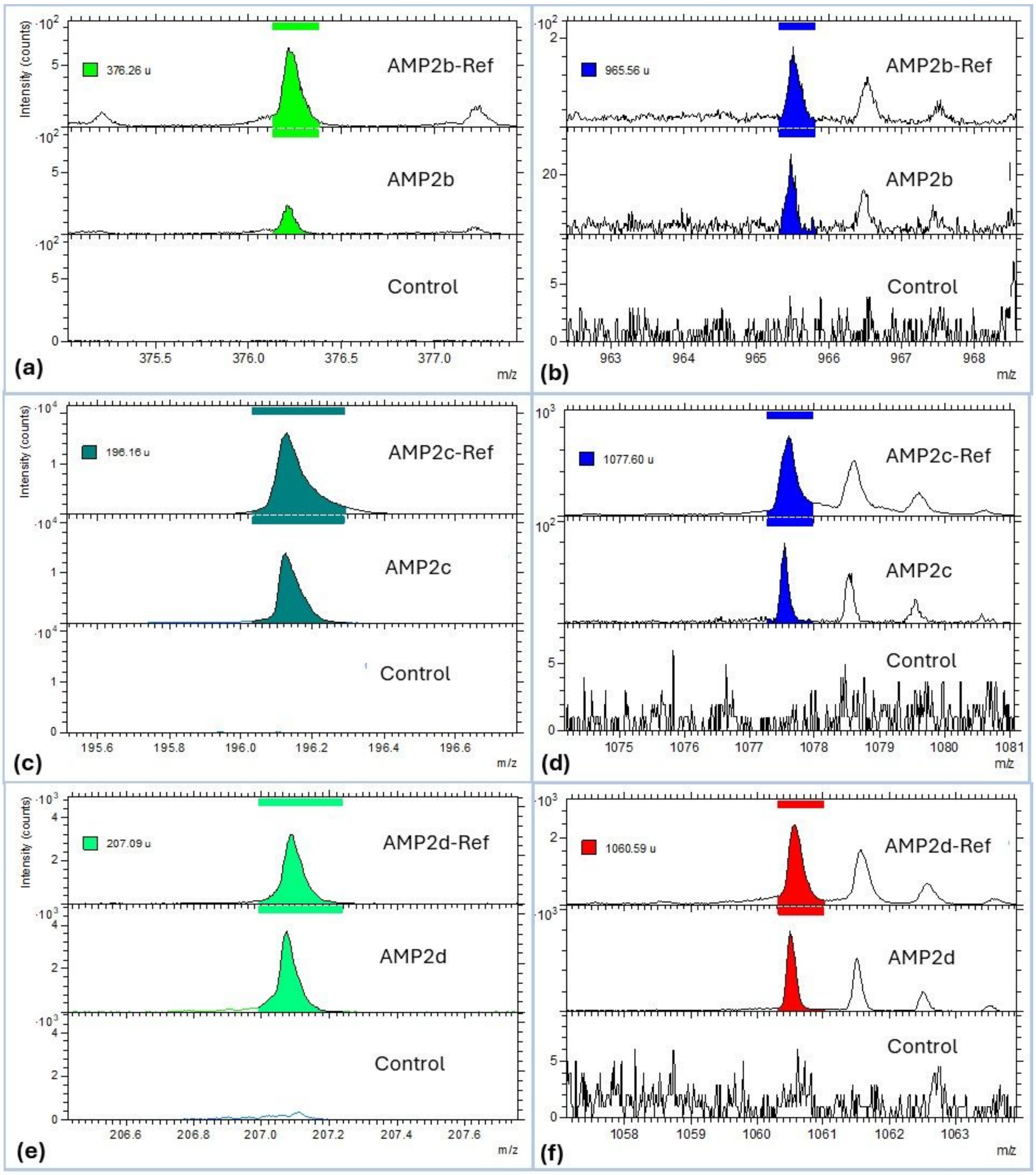

Figure S5: Examples of peptide fragments generated during ToF-SIMS analysis of peptides 2b, 2c and 2d. (**a, c, e**) Graphs show mass spectra of lower molecular mass peptide fragments (~200-400 u). (**b, d, f**) Graphs show mass spectra of higher molecular mass fragments (~1000 – 1100 u). Each graph shows the mass spectrum of the non-conjugated peptide (top), conjugated peptide via click reaction (middle), and a control surface in which Cu^{2+} was omitted during the click reaction (bottom)

Fragments to measure were selected so that no interference from other surface constituents (i.e. glass, silane, AuNPs and PEG-alkyne spacer) that could be detected in the control existed.

Generally, the higher mass of the specific peptide fragment identified on the glass surface, the stronger is the validity of the identification. For example, in the case of peptide **2b** a fragment with mass 1459.81 u was detected on the glass surface. This fragment consists of almost the full peptide, which has a mass of 1462.53 u. For **2c** and **2d** mass fragments having the same mass as the entire peptide was found, which is indeed a very strong identification of peptide presence on the surface. Heat maps showing the flux of fragments of peptide **2d** normalized to total ion flux and are presented in Figure 4 of the main article. Figure S6 shows the corresponding data obtained for peptide **2b** and **2c**.

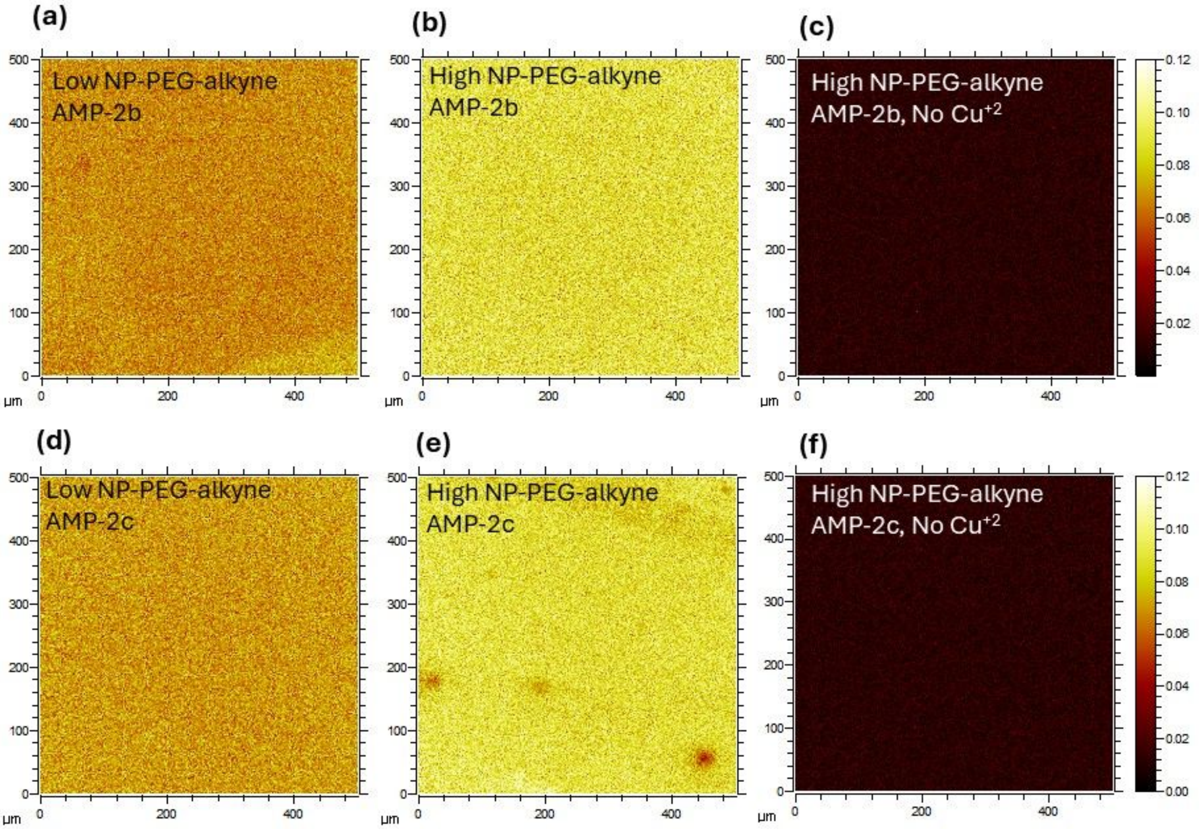

Figure S6: ToF-SIMS heatmaps showing the distribution of peptide 2b and 2c. (a) Peptide **2b** bound to a surface coated with low coverage of NP-PEG-alkyne. **(b)** Peptide **2b** bound to a surface coated with high coverage of NP-PEG-alkyne. **(c)** Peptide **2b** bound to a surface coated with high coverage of NP-PEG-alkyne in the absence of copper ions. **(d)** Peptide **2c** bound to a surface coated with low coverage of NP-PEG-alkyne. **(e)** Peptide **2c** bound to a surface coated with high coverage of NP-PEG-alkyne. **(f)** Peptide **2c** bound to a surface coated with high coverage of NP-PEG-alkyne in the absence of copper ions.

Localized Surface Plasmon Resonance (LSPR) for dynamic analysis of peptide attachment to Au-nanodisc functionalized with PEG-alkyne.

LSPR is a surface-sensitive method in which the change in resonance wavelength of the localized surface plasmon of circular Au-nanodiscs is measured in response to molecules binding to or assemble close to the disks' surfaces. This allows the detection of peptide binding, both due to adsorption and covalent attachment, to the SH-PEG-alkyne functionalized Au surface. In this study we used LSPR to study both the rate of binding to be sure we have reached saturation of peptide attachment, but also to demonstrate the covalent binding of the peptides to the surface. The covalent attachment was investigated by omitting Cu^{2+} during the click reaction. Representative graphs are shown in Figure S7.

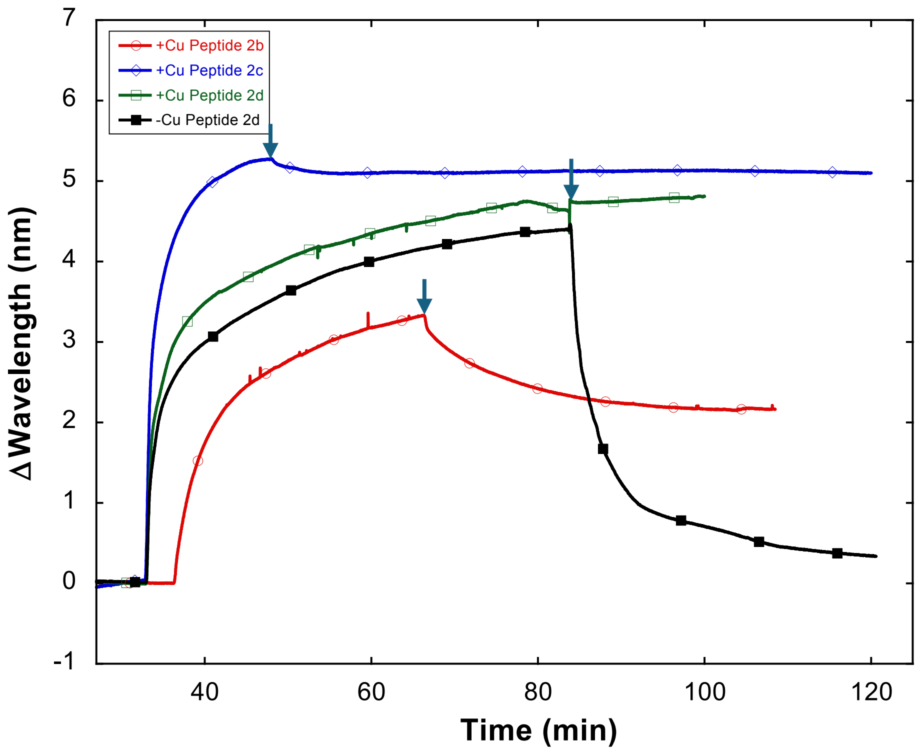

Figure S7: Representative LSPR graphs showing the shift in wavelength as a function of binding time. Open red circle = peptide **2b** with Cu^{2+} present in the click solution, open blue diamond = peptide **2c** with Cu^{2+} present in the click solution, open green rectangle = peptide **2d** with Cu^{2+} present in the click solution and filled black rectangle = peptide **2d** with no Cu^{2+} present in the click solution. The arrows mark the shift from click solution to wash solution (PBS).

As investigated by LSPR, the coupling of peptide to alkyne functionalized Au-nanodisc was efficient and saturation coverage was reached within 60 min from injection for all peptides. The kinetics of binding varied with peptide structure. The additional PEG chain of peptide **2b** decreased the rate of binding compared to peptide **2c**. The bulky PEG molecule induces some steric hinderance, which together with the entropic resistance has been attributed to decreased reactivity of functional end-groups on PEG chains (2). It can also be noted that the cyclic peptide **2d** showed a lower rate of binding compared to the linear counterpart peptide **2c**. This can be attributed to (1) cyclic molecules often have more restricted conformations, which can limit the accessibility of reactive sites. This makes it harder for the linear molecule to approach and react with the cyclic molecule (2) Cyclic molecules, especially smaller rings, can have

significant ring strain. This strain can affect the stability of the transition state during the reaction, potentially making the reaction less favorable (3) Cyclic molecules are more rigid compared to linear molecules, which can reduce the number of effective collisions between the reactants. This rigidity can slow down the reaction rate.

The click reaction was terminated by injection of PBS buffer (indicated by arrows in Figure S7). The washing procedure removed minor amounts of physiosorbed peptide **2c** and **2d** indicating that most of the peptide was covalently attached. For peptide **2b** a larger fraction of bound peptide was physiosorbed and a long washing time was necessary to reach stable conditions. We attribute also this effect to the PEGylation of peptide **2b**. Upon prolonged wash no further detectable removal of any of the peptides **2b**, **2c** or **2d** was observed indicating stable formation of peptide coating. When copper was omitted during the click reaction the covalent attachment of peptide was minimized as indicated by the rapid and extensive removal of physiosorbed peptide upon wash.

Dynamic light scattering (DLS) analysis of AuNP modifications with SH-PEG-alkyne and binding of cAMPs

The AuNPs to be analyzed with DLS were coated with alkyne-PEG-thiols with molecular weight 600 Da or 3400 Da as detailed in the Method section of the main article. DLS measurements were done using a Zetasizer NanoZS instrument (Malvern Panalytical Ltd, United Kingdom). The sample chamber was set to 25 °C and the sample was measured in back scattering mode at a 173° angle. For each data point data was collected for 1 min. The size of the AuNPs diluted in MQ water was measured before and after modification with the SH-PEG-alkyne molecules (Figure S8).

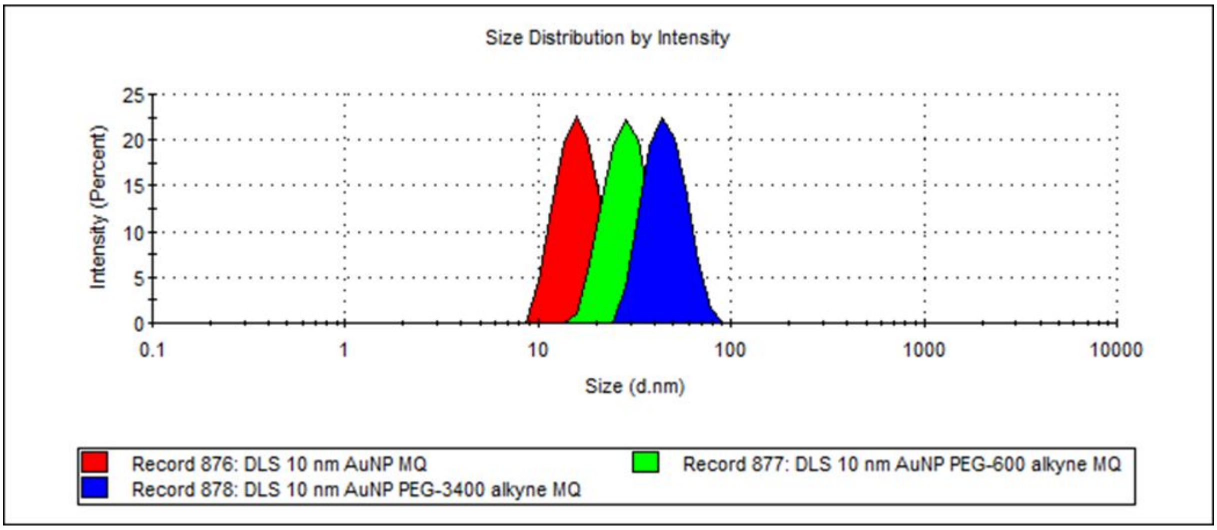

Figure S8: DLS data showing the size distribution of AuNPs before and after modification with SH-PEG in MQ water. The red peak shows the size distribution of uncoated AuNPs, the green peak shows the size distribution of AuNPs coated with SH-PEG-alkyne with MW 600 Da, the blue peak shows AuNPs coated with SH-PEG-alkyne with MW 3400 Da.

The DLS measurements indicated that the diameter of AuNPs before PEG coating was 17 nm. This is reasonable for AuNPs with core size 10 nm since DLS measures the hydrodynamic diameter of the AuNPs, which is typically up to 10 nm larger than the core diameter (3). The polydispersity index (PDI) of the unmodified AuNPs was 0.09, indicating a narrow size distribution. After modification with SH-PEG(600)-alkyne the particle diameter increased to 28 nm and the PDI increased to 0.13, and after modification with SH-PEG(3400)-alkyne the particle diameter increased to 46 nm and the PDI increased to 0.15. The increased PDI values may be due to the presence of a small fraction of aggregates forming during the modification and purification steps.

DLS experiments were also made in which the AuNP size was measured after binding of the three different cAMPs **2b**, **2c** and **2d** to the particles' surfaces. In these measurements, the PEG-coated AuNPs were added first to the click solution contained in a 1 mL cuvette to a final concentration of 3 nM, followed by the addition of copper and finally the different antimicrobial peptides (dissolved in DMSO) to a final concentration of 10 µM corresponding to approximately 10 peptides per available alkyne. A pipette was used to quickly mix the solution whereupon the cuvette was immediately put into the instrument and the measurements were started. Data was collected for 30 minutes, each data point corresponding to the average of 6

measurements each lasting 10 seconds. Figure S9 and Figure S10 show how the average AuNP sizes change over time for AuNPs modified with SH-PEG(600)-alkyne and SH-PEG(3400)-alkyne, respectively. The presented curves are the average values of three independent experiments.

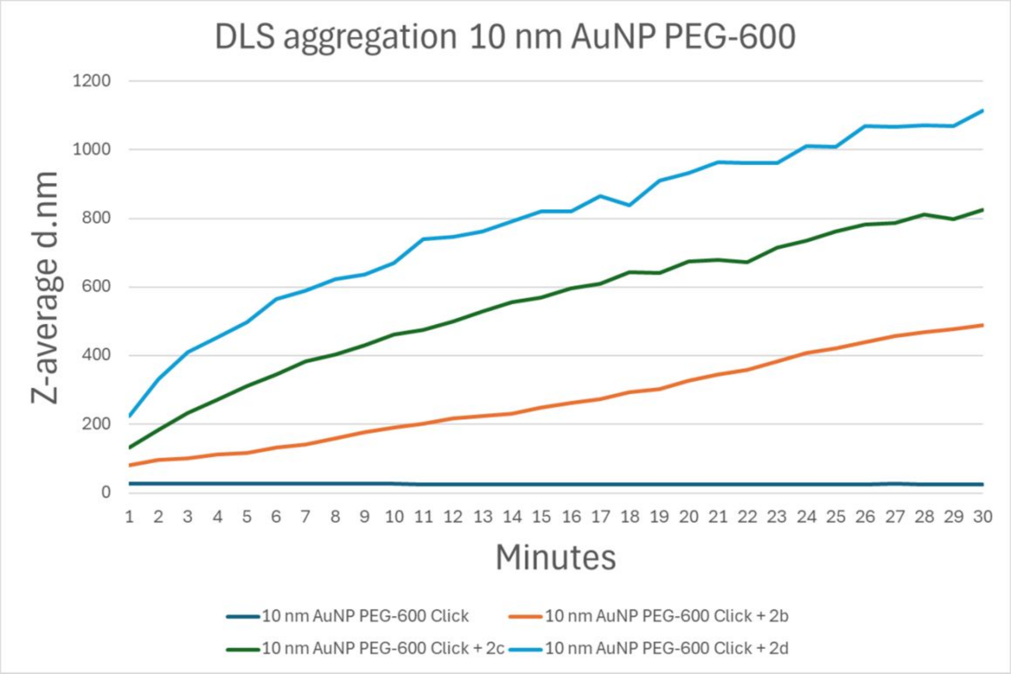

Figure S9: DLS data showing the Z-average of AuNPs coated with SH-PEG600-alkyne contained in a click reaction solution (dark blue) after the addition of cAMPs **2b** (orange), **2c** (green) and **2d** (light blue).

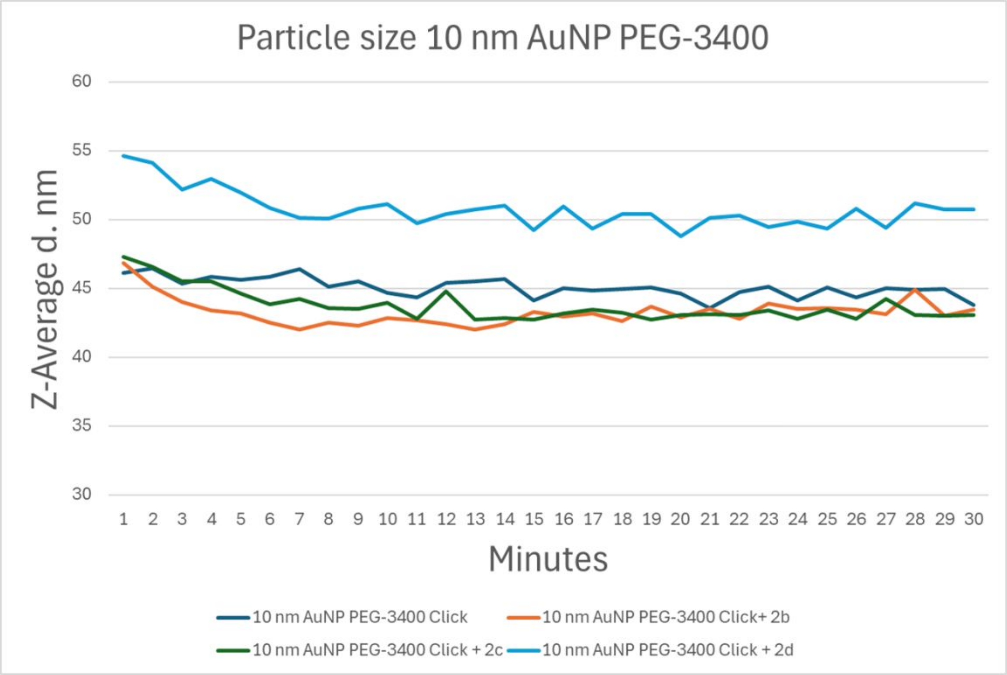

Figure S10: DLS data showing the Z-average over time of AuNPs coated with SH-PEG3400-alkyne contained in a click reaction solution (dark blue) after the addition of cAMPs **2b** (orange), **2c** (green) and **2d** (light blue).

Both the AuNPs coated with SH-PEG(600)-alkyne and with SH-PEG(3400)-alkyne remained stable (did not change size) when dissolved in the click reaction solution. Upon addition of the cAMPs, the AuNPs coated with SH-PEG(600)-alkyne immediately started to aggregate. The initial aggregation phase was too fast to be measured, but from the data acquired during the first five minutes of the experiment the addition of peptide **2d** led to approximately twice as fast aggregation as **2c** and five times faster aggregation than peptide **2b**. In contrast, the AuNPs coated with SH-PEG(3400)-alkyne remained, essentially, unaltered upon addition of the peptides. Only for **2d** a small increase of the average particle size was detected. Since this increase was accompanied by an increase of the PDI to 0.33 we interpret the size increase as due to the formation of a sub-fraction of aggregated particles.

References

(1) Lundgren, A. O.; Björefors, F.; Olofsson, L. G. M.; Elwing, H., Self-Arrangement Among Charge-Stabilized Gold Nanoparticles on a Dithiothreitol Reactivated Octanedithiol Monolayer. *Nano Lett* **2008,** *8* (11), 3989-3992.

(2) Sahoo, B., et al. Influence of PEG endgroup and molecular weight on its reactivity for lipase-catalyzed polyester synthesis. *Biomacromolecules* **2006**, *7* (4), 1042-1048.

(3) Agnarsson, B., et al. Evanescent Light-Scattering Microscopy for Label-Free Interfacial Imaging: From Single Sub-100 nm Vesicles to Live Cells. *ACS Nano* **2015**, *9* (12), 11849-11862.
